# Decoding expectation and surprise in dementia: the paradigm of music

**DOI:** 10.1093/braincomms/fcab173

**Published:** 2021-08-10

**Authors:** Elia Benhamou, Sijia Zhao, Harri Sivasathiaseelan, Jeremy C S Johnson, Maï-Carmen Requena-Komuro, Rebecca L Bond, Janneke E P van Leeuwen, Lucy L Russell, Caroline V Greaves, Annabel Nelson, Jennifer M Nicholas, Chris J D Hardy, Jonathan D Rohrer, Jason D Warren

**Affiliations:** 1 Dementia Research Centre, UCL Queen Square Institute of Neurology, University College London, London WC1N 3AR, UK; 2 Department of Experimental Psychology, University of Oxford, Oxford OX2 6GG, UK; 3 Department of Medical Statistics, Faculty of Epidemiology and Population Health, London School of Hygiene and Tropical Medicine, London, UK

**Keywords:** music, surprise, frontotemporal dementia, pupillometry, voxel-based morphometry

## Abstract

Making predictions about the world and responding appropriately to unexpected events are essential functions of the healthy brain. In neurodegenerative disorders, such as frontotemporal dementia and Alzheimer’s disease, impaired processing of ‘surprise’ may underpin a diverse array of symptoms, particularly abnormalities of social and emotional behaviour, but is challenging to characterize. Here, we addressed this issue using a novel paradigm: music. We studied 62 patients (24 female; aged 53–88) representing major syndromes of frontotemporal dementia (behavioural variant, semantic variant primary progressive aphasia, non-fluent-agrammatic variant primary progressive aphasia) and typical amnestic Alzheimer’s disease, in relation to 33 healthy controls (18 female; aged 54–78). Participants heard famous melodies containing no deviants or one of three types of deviant note—acoustic (white-noise burst), syntactic (key-violating pitch change) or semantic (key-preserving pitch change). Using a regression model that took elementary perceptual, executive and musical competence into account, we assessed accuracy detecting melodic deviants and simultaneously recorded pupillary responses and related these to deviant surprise value (information-content) and carrier melody predictability (entropy), calculated using an unsupervised machine learning model of music. Neuroanatomical associations of deviant detection accuracy and coupling of detection to deviant surprise value were assessed using voxel-based morphometry of patients’ brain MRI. Whereas Alzheimer’s disease was associated with normal deviant detection accuracy, behavioural and semantic variant frontotemporal dementia syndromes were associated with strikingly similar profiles of impaired syntactic and semantic deviant detection accuracy and impaired behavioural and autonomic sensitivity to deviant information-content (all *P* < 0.05). On the other hand, non-fluent-agrammatic primary progressive aphasia was associated with generalized impairment of deviant discriminability (*P* < 0.05) due to excessive false-alarms, despite retained behavioural and autonomic sensitivity to deviant information-content and melody predictability. Across the patient cohort, grey matter correlates of acoustic deviant detection accuracy were identified in precuneus, mid and mesial temporal regions; correlates of syntactic deviant detection accuracy and information-content processing, in inferior frontal and anterior temporal cortices, putamen and nucleus accumbens; and a common correlate of musical salience coding in supplementary motor area (all *P* < 0.05, corrected for multiple comparisons in pre-specified regions of interest). Our findings suggest that major dementias have distinct profiles of sensory ‘surprise’ processing, as instantiated in music. Music may be a useful and informative paradigm for probing the predictive decoding of complex sensory environments in neurodegenerative proteinopathies, with implications for understanding and measuring the core pathophysiology of these diseases.

## Teaser

Benhamou et al. report that major dementias have distinct behavioural, autonomic and neuroanatomical profiles of musical ‘surprise’ processing. While Alzheimer’s disease patients showed normal reactivity to deviants in familiar melodies, frontotemporal dementia patients had specific impairments, suggesting that musical ‘tools’ could measure complex behavioural changes in these diseases.

## Graphical Abstract

**Graphical Abstract:**
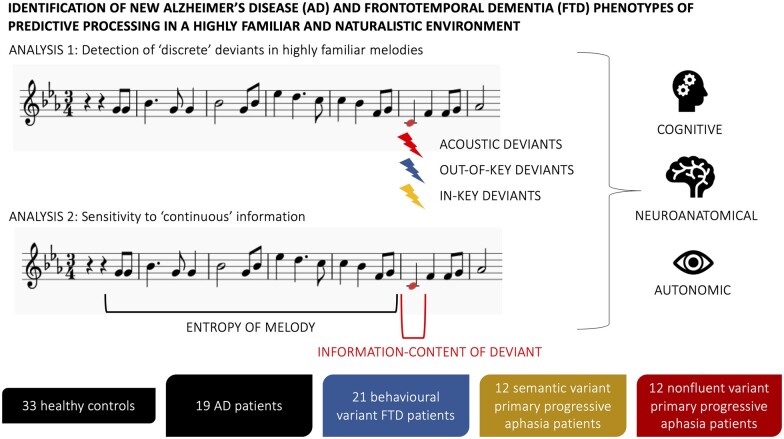

## Introduction

Predicting the future based on past experience and responding appropriately to unexpected, ‘surprising’ events are fundamental functions of the healthy brain. These functions are targeted prominently and early in a number of neurodegenerative disorders. Impaired understanding of social norms, ‘rules’ and boundaries define behavioural variant frontotemporal dementia (bvFTD),^1–3^ signifying deficient predictive ‘modelling’ of the socio-emotional milieu^4^ and implicating more fundamental processes, such as error detection and monitoring, ambiguity and conflict resolution, probabilistic learning, risk evaluation and decision making.^1^^,^^5–11^ These processes are also affected in other FTD syndromes—semantic variant primary progressive aphasia (svPPA) and non-fluent-agrammatic (nfv) PPA—and Alzheimer’s disease.^6^^,^^7^^,^^11–20^

A plausible unifying mechanism for this diverse phenotypic spectrum is impaired integration of expectation (established by environmental context) with surprise (unexpected events). This mechanism has a neurophysiological signal in altered mismatch negativity^21–27^ and a neuroanatomical signature in dysfunctional frontotemporal neural circuitry.^21^^,^^22^^,^^28–32^ Impaired deviance detection may develop early in the course of neurodegeneration and indexes a core mechanism of the culprit proteinopathy in animal models.^33–35^ Taken together, this evidence suggests that neural processes establishing expectations and decoding ‘surprise’ may underpin diverse pathophysiological and clinical effects of dementias, such as FTD and Alzheimer’s disease. However, these processes remain poorly characterized and difficult to quantify, particularly in the setting of neurodegenerative disease.

As a paradigm for addressing these issues, music is particularly promising. Music is ubiquitous in daily life and constitutes a model ‘environment’ that is bound by a finite set of implicit ‘rules’. Even musically untrained listeners internalize these automatically through lifelong exposure to the dominant musical culture and accordingly acquire strong and reliable psychological expectations about musical patterns and events.^36^ Moreover, ‘surprise’ of various kinds is easily engendered in music by violating these rules. The inherently rule-based nature of music and the role of expectation and surprise in achieving many of its psychological and physiological effects^37^^,^^38^ together suggest that the neural processing of music might follow the principles of ‘predictive coding’. Predictive coding has gained wide currency as a paradigm of brain operation and in particular, the role of active neural inference in making and iteratively refining predictions about the environment, thereby minimizing error and optimizing neural representations of the world at large.^39^^,^^40^ The probabilistic structure of musical sequences (melodies) can be analysed using information-theoretic approaches that quantify both the listener’s degree of uncertainty about the prevailing musical environment (its entropy or unpredictability) and the information-content (IC; degree of unexpectedness or ‘surprise’) associated with a particular musical event (such as a deviant note).^41^

Our emerging picture of the musical brain, derived from cognitive and functional neuroimaging studies in both health and disease,^42^ provides candidate neural substrates for predictive coding of musical information. In the healthy brain, a parametric correlate of musical note unexpectedness (high IC) has been identified in anterior cingulate and insula^43^ and neural correlates of processing surprise and prior uncertainty in amygdala, hippocampus and nucleus accumbens.^44^ Canonical syndromes of FTD and Alzheimer’s disease have distinctive profiles of musical perceptual, semantic and affective impairment^42^^,^^45–49^ that reflect the targeting of these distributed networks by neurodegenerative proteinopathies. Dementias are therefore anticipated to produce separable but overlapping cognitive and physiological profiles of musical ‘surprise’ processing. Further, processing of higher order spectrotemporal statistics of speech signals is affected in nfvPPA and svPPA.^50^^,^^51^ A predictive coding account of the impaired understanding of degraded speech has been developed for nfvPPA^52^ while changes in the macro- and meso-scale functional organization of neural circuits that support predictive coding have been described in bvFTD.^22^^,^^29^^,^^32^ Moreover, the processing of music and social and emotional signals share key neural resources that are targeted in FTD and Alzheimer’s disease.^42^^,^^53^^,^^54^ However, music remains unexplored as a paradigm of sensory ‘surprise’ processing (and by extension, complex behavioural alterations) in neurodegenerative disease.

Here, we addressed this issue in a cohort of patients representing major FTD syndromes—bvFTD, svPPA and nfvPPA—and typical Alzheimer’s disease, referenced to healthy older individuals. We manipulated musical surprise—in both its traditional psychological and information-theoretic senses—by inserting notes representing three kinds of deviant ‘event’ condition into an ‘environment’ of familiar melodies with variable predictability levels. These deviant conditions targeted three levels of musical organization: basic acoustic structure, the general harmonic rules governing musical sequences or musical ‘syntax’ and the regularities or ‘semantics’ specifying melodies as individual musical objects.^55^ We simultaneously assessed performance at deviant detection and pupillary responses to deviant notes. Pupil dilatation, as a marker of physiological arousal potentially dissociates from cognitive processing and robustly signals violation of expectations and statistical regularities of sensory input in the healthy brain.^56–61^ We estimated the information-theoretic properties of our musical stimuli using a computational model of musical informational dynamics,^62^ in order to assess how environmental predictability (entropy) and the sensory informational structure of deviant ‘surprising’ events related to behavioural and physiological responses in different dementia syndromes. Structural neuroanatomical associations of behavioural reactivity to melodic deviants were assessed using voxel-based morphometry (VBM) of patients’ brain MRI.

Based on previous work addressing more general musical and auditory cognitive and perceptual functions in dementia,^45–49^^,^^63–65^ we hypothesized that FTD syndromes would be associated with more severe impairments of musical deviant detection and autonomic reactivity than would Alzheimer’s disease; and further, that FTD syndromes would be stratified by their relative degree of impairment in processing particular deviant conditions: impaired detection of semantic and syntactic deviants in bvFTD and svPPA^45^^,^^49^ and a generalized impairment of auditory deviant detection in nfvPPA.^50^^,^^66^ We additionally hypothesized that sensitivity to information-theoretic parameters of melodies (deviant surprise, melody entropy) would be relatively more severely reduced in bvFTD and svPPA than in other participant groups.^8^^,^^22^^,^^32^^,^^50^^,^^51^ Finally, we hypothesized that the cognitive coding of musical surprise in the patient cohort would have separable neuroanatomical correlates within the hierarchical distributed brain networks previously implicated in processing different kinds of musical information.^42^^,^^43^^,^^49^^,^^67–72^

## Methods and materials

### Participants

Nineteen patients with typical Alzheimer’s disease, 21 with bvFTD, 12 with svPPA and 12 with nfvPPA were recruited. All patients fulfilled consensus clinical criteria for their syndromic diagnosis,^73–75^ of mild to moderate severity. Thirty-three healthy older individuals with no history of neurological or psychiatric disorders also participated. None of the participants had a history of clinically relevant hearing loss, congenital amusia or pupillary disease. All participants had a comprehensive general neuropsychological assessment.

To index participants’ musical experience, patients’ caregivers and healthy control participants completed a questionnaire detailing years spent learning or playing an instrument (prior musical expertise, scored on a 4-point scale ranging from 0 (never played an instrument or sang in a choir) to 3 (learned an instrument for >10 years) and hours per week on average currently spent listening to music.^76^ All participants had audiometric screening of peripheral hearing function (indexed as a mean hearing threshold via the better ear over 0.5, 1, 2, 4 and 8 kHz) and assessment of their ability to discriminate the direction of pitch changes relevant to melodies. Details of these procedures are in Supplementary material. Demographic details (including musical background), clinical and general neuropsychological characteristics of the study cohort are summarized in Table 1.

**Table 1:** Demographic, clinical and general neuropsychological characteristics of participant groups

Characteristics	Controls	AD	bvFTD	svPPA	nfvPPA
Demographic and clinical					
No. (male:female)	15:18	9:10	**16:5**	7:5	8:4
Age (years)	64.8 (5.1)	**71.4 (8.6)^a^**	65 (5.7)	65.5 (6.0)	70.6 (7.3)
Handedness (R:L)	31:2	17:2	19:2	12:0	12:0
Education (years)	15.9 (2.3)	15.1 (2.9)	14.6 (2.9)	15 (2.6)	13.2 (2.6)
Symptom duration (years)	N/A	6.1 (4.0)	8.5 (4.5)	5.9 (2.2)	3.7 (1.8)^a^
MMSE (/30)	29.4 (0.9)*	**21.1 (5.5)**	25.9 (3.4)	**23 (7.4)**	**20 (8.4)^a^**
Hearing threshold (dB)^b^	27.6 (9.4)	31.6 (6.6)	28.5 (5.6)	25.7 (8.1)	32.8 (7.5)
Genetic mutations	N/A	0	4 *C9orf72*, 5 *MAPT*	0	2 *GRN*
Acetylcholinesterase inhibitor use (*n*)	N/A	11	0	0	0
Neuropsychological functions					
Episodic memory					
RMT words (/50)	40.9 (6.3)**	**31.7 (6.9)**	**34.3 (7.6)**	**30.7 (3.4)^c^**	**35.4 (5.6)**
RMT faces (/50)	48 (2.3)**	**30.9 (7.5)**	**38.6 (7.7)**	**34.8 (6.5)^c^**	**37.9 (9.5)**
Camden PAL (/24)	20.2 (2.0)**	**6.8 (5.4)^c^**	**11.1 (7.2)**	**9.4 (8.1)**	**14.4 (5.0)**
Executive skills					
WASI block design (/71)	51 (12.7)***	**15.1 (8.5)^b^^,d^**	**31.2 (13.4)**	41.1 (17.9)	**19.3 (20.4)^b^^,d^**
WASI matrices (/32)	25.5 (5.2)***	**13.2 (4.7)^d^**	**17.3 (7.2)^d^**	26.4 (5.0)	**16.6 (9.7)^d^**
WMS-R digit span forward (max)	7.1 (1.2)***	**6.2 (1.2)**	**6.2 (1.2)**	6.5 (1.1)	**4.6 (1.3)^b^^,d,e^**
WMS-R digit span reverse (max)	6 (1.1)***	**3.8 (1.5)^d^**	**4.6 (1.5)**	5.4 (1.6)	**3.4 (0.8)^d^**
D-KEFS stroop colour naming (s)	28.4 (4.8)**	**52.5 (14.2)^b^**	**44.1 (15.9)**	**43.2 (14.9)**	**77.7 (14.9)^b^^,d,e^**
D-KEFS stroop word reading (s)	21.7 (4.5)**	**36.4 (16.9)^b^^,d^**	**26.2 (6.0)**	**27.6 (10.2)**	**68.7 (20.5)^b^^,d,e^**
D-KEFS stroop interference (s)	56 (15.8)**	**126.3 (38.8)^b^^,d^**	**78.9 (24.9)**	**83.3 (26.9)**	**139.6 (24.3)^b^^,d^**
Trails A (s)	28.5 (10.6)**	**85.6 (40.2)^b^^,c,d^**	**48 (21.2)**	**45.4 (18.1)**	**60.1 (43.2)**
Trails B (s)	65.2 (23.7)**	**219.5 (83.1)^d^**	**157.3 (90.4)**	**134.4 (97)**	**147.2 (64.7)**
Letter fluency (F, 1 min)	17.9 (5.5)**	**9.7 (4.3)**	**10.6 (5.9)**	**8.1 (4.6)**	**6.6 (6.3)**
Category fluency (animals, 1 min)	25 (4.7)**	**10.8 (4.7)**	**13.5 (6.6)**	**6.1 (5.6)^b^**	**10.1 (5.3)**
Language skills					
WASI vocabulary (/80)	71.6 (5.2)**	**56.8 (13.1)**	**47.9 (19.9)**	**27 (21.4)^b^^,e^**	**24 (21.4)^b^^,e^**
Graded naming test (/30)	24.9 (3.0)**	**14.6 (8.3)**	**14.8 (9.9)**	**1.9 (5.3)^b^^,c,e^**	**14.9 (7.7)**
BPVS (/150)	147.9 (1.2)***	**133.4 (24.2)**	**115.7 (44.9)**	**64.6 (46.1)^b^^,e^**	**105.3 (48.8)**
PALPA 55 (/24)	23.7 (0.6)	N/A	N/A	**21 (2.9)**	**16.6 (4.4)d**
Other skills					
Graded difficulty arithmetic (/24)	17 (4.2)**	**5.4 (5.8)^d^**	**8.7 (6.7)**	13.4 (5.8)	**6.4 (6.7)**
VOSP object decision (/20)	18.5 (1.7)**	**10.6 (5.1)^b^**	**15.1 (5.2)**	**11.5 (6.2)**	**14.3 (6.6)**
Musical skills and background					
Pitch direction discrimination (/10)^a^	9.1 (1.1)	8.6 (1.3)	**7.9 (1.5)^c^**	9.6 (0.5)	8.3 (2.2)
Recognition of melodies: % correct	0.94 (0.08)	0.93 (0.08)	**0.85 (0.16)**	**0.89 (0.04)**	0.84 (0.17)
*d*-Prime	3.25 (0.54)	**2.56 (0.60)**	**2.16 (1.07)**	**1.95 (1.61)**	2.61 (1.10)
Prior musical expertise (/3)^f^	0.8 (0.9)	0.8 (0.6)	1 (0.8)	0.9 (1.0)	0.5 (0.7)
Current music listening (h/week)	7.1 (7.6)	5.8 (5.7)	7.6 (6.6)	7.7 (10.5)	5.2 (5.5)

Mean (standard deviation) scores are shown unless otherwise indicated; maximum scores are shown after tests (in parentheses). Significant differences (*P* < 0.05) from healthy control values are indicated in bold; *data from 19 healthy controls; **data from 23 healthy controls; ***data from 25 healthy controls.

AD, patient group with typical Alzheimer’s disease; BPVS, British Picture Vocabulary Scale; bvFTD, patient group with behavioural variant frontotemporal dementia; *C9orf72*, pathogenic mutation in open reading frame of chromosome 9; Category fluency totals for animal category and letter fluency for the letter F in 1 min; Controls, healthy control group; D-KEFS, Delis Kaplan Executive System; Graded Difficulty Arithmetic test; Graded Naming Test; *GRN*, pathogenic mutation in progranulin gene; *MAPT*, pathogenic mutation in microtubule-associated protein tau gene; MMSE, Mini-Mental State Examination score; N/A, not assessed; nfvPPA, patient group with non-fluent-agrammatic variant primary progressive aphasia; PAL, Paired Associate Learning test; PALPA 55, Psycholinguistic Assessments of Language Processing in Aphasia subtest for Auditory Sentence Comprehension; RMT, Recognition Memory Test; svPPA, patient group with semantic variant primary progressive aphasia; Trails-making scores based on maximum time achievable of 2.5 min on task A and 5 min on task B; VOSP, Visual Object and Spatial Perception Battery—Object Decision test; WASI, Wechsler Abbreviated Scale of Intelligence; WMS, Wechsler Memory Scale.

a See text and Supplementary material for details.

b Significantly lower than bvFTD group.

c Significantly lower than nvfPPA group.

d Significantly lower than svPPA group.

e Significantly lower than AD group.

Musical training assessed on a four-point scale: (0: never played an instrument or sang in a choir, to 3: learned an instrument for >10 years; all participants were lifelong British residents sharing a similar socio-economic and musical milieu).

All participants gave informed consent for their involvement in the study. Ethical approval was granted by the University College London and National Hospital for Neurology and Neurosurgery Joint Research Ethics Committees in accordance with Declaration of Helsinki guidelines.

### Experimental design and stimuli

Based on a survey of 15 older British individuals who did not participate in the main study, 48 melodies rated as highly familiar on a 5-point scale [ranging from 1 (completely unfamiliar) to 5 (very familiar)] were selected and all transcribed for solo piano (details of all stimuli in Supplementary Table 1; stimulus examples in Supplementary material). From these famous melodies, we created four experimental conditions, comprising melodies containing either: no deviant note; a single note that deviated from the canonical melody while preserving its key (‘semantic’ deviants); a single note that deviated from the canonical melody by violating its key or tonality (‘syntactic’ deviants); or a single note that deviated in timbre (‘acoustic’ deviants, formed from white-noise bursts). The final stimulus set comprised 48 trials (12 exemplars of each condition).

### Experimental procedure

All participants were first familiarized with the experiment to ensure they understood and could comply with the procedure. The participant was seated fixating a desktop computer monitor in a dimly and uniformly illuminated, quiet room. Pupil area was measured continuously using an infrared camera (details in Supplementary material). Stimuli were presented in randomized order from a notebook computer running Experiment Builder^®^ (www.sr-research.com/experiment-builder) binaurally via headphones at a constant, comfortable listening level. The task on completion of each trial was to decide whether the melody contained a ‘wrong note’.

Following the pupillometry session, we conducted a test to assess each participant’s recognition of stimulus melodies, based on a two-alternative forced-choice decision between famous tunes (the pupillometry stimuli) and newly composed melodies (details in Supplementary material).

All participant responses were stored for offline analysis; no feedback about performance was given and no time limits on responses were imposed.

### Analysis of behavioural data

All behavioural data were analysed using Stata14.1^®^. Demographic characteristics and neuropsychological data were compared between participant groups using chi-squared tests for categorical variables, and for continuous variables, either one-way ANOVA followed by two-sample *t*-tests or non-parametric Kruskal–Wallis tests followed by Wilcoxon rank-sum tests when *t*-test assumptions were violated. *d*-Prime values were calculated as a measure of each participant’s sensitivity for detection of deviants in each condition. Restricted maximum likelihood mixed effects’ models, with participant identity as a random effect, were used to analyse *d*-prime (discriminability) and hit rate (deviant detection accuracy). Joint Wald tests of the relevant coefficients were used to examine the main effects of diagnostic group and experimental condition (deviant type) and their interaction. *Post-hoc* pairwise group comparisons were performed where main effects were found. To take account of potentially confounding factors, age, gender, pitch direction discrimination performance (indexing pitch contour perception, musical working memory and task decision-making), prior musical expertise score and mean hours of listening to music per week were included as covariates in the regression model. In addition, we used Pearson’s correlation tests to separately assess any relationship between *d*-primes with age, gender, prior musical expertise and hours of listening, ability to recognize familiar melodies, peripheral hearing function, pitch contour processing (pitch direction discrimination score) and general disease factors [Mini-Mental State Examination (MMSE) score, symptom duration], across the patient cohort.

In order to examine the sensitivity and specificity of deviant detection profiles for predicting disease, we conducted a receiver-operating-characteristic (ROC) analysis and calculated area under the curve (AUC) coefficients assessing how well melodic deviant detection rate (*d*-primes) in each experimental condition discriminated syndromic groups from the healthy control group (further details are in Supplementary material).

A threshold *P* < 0.05 was accepted as the criterion of statistical significance for all tests.

### Analysis of pupillometric data

Pupil diameter data were pre-processed using previously described procedures^77^ (details in Supplementary material), normalized to baseline and time-domain-averaged across trials for each condition. To identify time windows with significant discrepancies between deviant and ‘standard’ time series, a non-parametric bootstrap-based statistical analysis was used^78^ (10 000 iterations; with replacement) with family-wise error (FWE)-corrected cluster-size threshold *P* < 0.05.

To assess effects of participant group and experimental conditions on pupillary response magnitude, we extracted the maximum of the normalized pupil dilatation response (averaged across trials for each participant) during the 2 s following deviant onset, an interval chosen based on both the permutation analysis and previous studies.^79^ We used the same linear mixed model employed for the behavioural data (with the same covariates) to compare pupil response amplitudes between participant groups and experimental conditions.

### Information-theoretic modelling

To quantify the extent to which behavioural and autonomic responses were related to the level of predictability and surprise in the musical environment, an unsupervised machine-learning model of auditory expectancy (Information Dynamics of Music model^80^) was used to quantify the predictability of each note within our experimental melody stimuli (further details in Supplementary material and Supplementary Fig. 1). For each melody containing a pitch deviant (or, for melodies without deviants, equivalently positioned ‘standard’ notes), we calculated two key information-theoretic parameters: the IC of the deviant note and the mean entropy (ENT) of the melody stem (from the first note to the note immediately preceding the deviant note; see details and Supplementary Table 1 in Supplementary material). The IC parameter allowed us to determine whether deviant detection accuracy and pupillary reactivity were related to deviant unexpectedness. The ENT parameter allowed us to determine the effect of melody (musical environmental) predictability, for melodies categorized as more predictable (low ENT) or less predictable (high ENT) relative to the median ENT value for the entire stimulus set.

For each participant group, we assessed correlations of stimulus IC score with trial-by-trial deviant detection accuracy (hit rate) and maximum pupillary dilatation response (during the 2 s following deviant onset) over the combined melody set and separately for each melody ENT category, using non-parametric Spearman’s correlation tests. We compared correlation strengths between participant groups using one-tailed *z*-tests, after transforming correlations to Fischer *z*-scores.^81^ A threshold *P* < 0.05 was accepted as the criterion of statistical significance for all tests.

### Brain image acquisition and analysis

For 58 patients (15 Alzheimer’s disease, 20 bvFTD, 12 svPPA, 11 nfvPPA), T1-weighted volumetric brain MRI were acquired on a Prisma 3 T MRI scanner using a 32-channel phased-array head-coil and pre-processed using standard procedures in SPM12 (www.fil.ion.ucl.ac.uk/spm^82^; details in Supplementary material).

In a VBM analysis of the combined patient cohort, full factorial linear regression models with diagnostic group as the main factor were used to assess associations between regional grey matter volume (indexed as voxel intensity) separately with detection accuracy for each deviant condition and with Spearman’s rho values for the correlation between detection accuracy and deviant IC. Age, total intracranial volume and pitch direction score were incorporated as covariates of no interest. Statistical parametric maps of regional grey matter associations were generated using an initial peak voxel and cluster-forming uncorrected threshold *P* < 0.001 and evaluated at peak voxel statistical significance level *P* < 0.05 after FWE correction for multiple voxel-wise comparisons within pre-specified anatomical regions of interest (see Supplementary Fig. 2). These regions were informed by previous studies of music and musical surprise processing in both the healthy brain and neurodegenerative disease and comprised: a posterior temporo-parietal network involved in sensory pattern analysis (posterior superior temporal gyrus, angular and supramarginal gyri and precuneus^65^^,^^83^^,^^84^) an anteroventral network involved in semantic appraisal (anterior superior temporal gyrus, middle temporal gyrus, temporal pole and inferior frontal gyrus^48^^,^^49^^,^^65^^,^^71^^,^^85^^,^^86^) a striato-limbic network involved in emotion and reward evaluation (putamen, caudate, nucleus accumbens, hippocampus and amygdala^44^^,^^45^^,^^70^^,^^87–89^) and a cingulo-insular network involved in salience processing and motor output (anterior insula, anterior cingulate cortex and supplementary motor area^90–92^).

### Data availability

We are precluded by institutional ethics agreements from publishing the full dataset in the public domain. However, data and stimuli will be made available on reasonable request to Prof Warren, under appropriate data transfer agreements.

## Results

### General participant characteristics

Patient groups did not differ significantly from healthy controls in gender distribution, handedness, years in formal education or composite audiometry score (all *P* > 0.05; Table 1). Patients with Alzheimer’s disease were on average significantly older than healthy controls (*P* < 0.05). The patient groups did not differ in mean symptom duration but did differ in overall severity of cognitive impairment (MMSE score; Kruskal–Wallis *H* = 102.9, *P* < 0.001). Participant groups did not differ significantly in musical expertise or average time spent listening to music each week (all *P* > 0.05; Table 1). However, the bvFTD group performed significantly worse than both the healthy control and svPPA groups on the pitch direction discrimination test (Kruskal–Wallis *H* = 10.9, *P* < 0.05 and *post-hoc* two-sample Wilcoxon tests, *P* < 0.05).

### Behavioural data: detection of deviants in melodies

**Figure 1:**
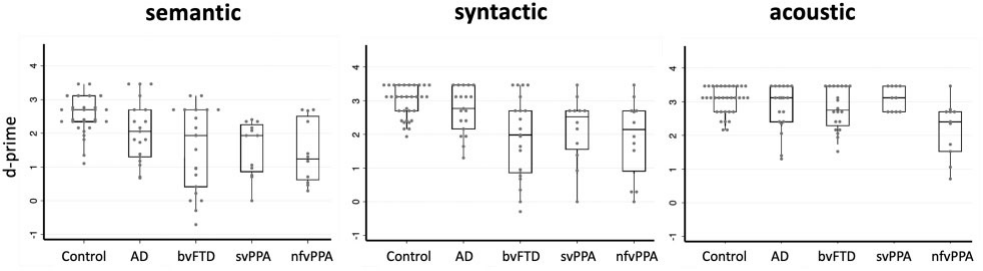
**Performance of detection of different types of deviants in melodies, for all participant groups.** For each panel (deviant condition), *d*-prime value is plotted for individuals within each participant group (see also Table 2). Each dot corresponds to an individual data point; boxes code the interquartile range, whiskers represent the ranges for the bottom 25% and the top 25% of the data values (excluding outliers) and the horizontal line in each box represents the median *d*-prime value. AD, patient group with Alzheimer’s disease; bvFTD, patient group with behavioural variant frontotemporal dementia; Control, healthy control group; nfvPPA, patient group with non-fluent-agrammatic variant primary progressive aphasia; svPPA, patient group with semantic variant primary progressive aphasia.

Group data for detection of deviants in melodies (hit rate and *d*-primes) are summarized in Table 2 and Supplementary Table 2. Individual *d*-primes data are plotted in Fig. 1. Results of detection accuracy (hit rate) are reported in Table 2.

**Table 2:** Summary of melodic deviant detection accuracy and pupillary response profiles for participant groups and conditions

Group	Condition	Behavioural response			Pupil peak response	
		Detection accuracy^a^	*d*-Prime	IC correlate	Amplitude^b^	IC correlate
Controls	No deviant	0.90 (0.10)	NA	rho = 0.41 (0.14)^c^	0.35 (0.22)	rho = 0.56 (0.14)^c^
	Semantic	0.89 (0.10)	2.58 (0.55)		0.53 (0.26)	
	Syntactic	0.98 (0.05)	2.95 (0.47)		0.61 (0.25)	
	Acoustic	1 (0)	3.04 (0.41)		0.78 (0.26)	
AD	No deviant	0.87 (0.16)	NA	rho = 0.10 (0.16)	0.47 (0.25)	rho = 0.53 (0.15)^c^
	Semantic	0.75 (0.26)	2.08 (0.91)		0.53 (0.27)	
	Syntactic	0.94 (0.14)	2.72 (0.68)		0.71 (0.44)	
	Acoustic	0.97 (0.07)	2.82 (0.68)		0.81 (0.46)	
bvFTD	No deviant	0.85 (0.16)	NA	**rho = −0.16 (0.19)^d^**	0.27 (0.31)	rho = 0.20 (0.15)
	Semantic	**0.62 (0.35)**	**1.52 (1.24)**		0.39 (0.30)	
	Syntactic	**0.73 (0.35)**	**1.91 (1.21)^e^**		0.42 (0.28)	
	Acoustic	0.99 (0.05)	2.80 (0.62)		0.68 (0.38)	
svPPA	No deviant	0.89 (0.10)	NA	**rho = −0.43 (0.14)^c^^,d^**	0.29 (0.32)	**rho = 0.21 (0.16)**
	Semantic	**0.61 (0.31)**	**1.56 (0.81)**		0.32 (0.26)	
	Syntactic	**0.77 (0.32)**	**2.16 (0.99)^e^**		0.36 (0.29)	
	Acoustic	1 (0)	3.09 (0.36)		0.71 (0.33)	
nfvPPA	No deviant	**0.74 (0.26)**	NA	rho = 0.39 (0.13)^c^	0.40 (0.31)	rho = 0.54 (0.11)^1^
	Semantic	0.71 (0.24)	**1.45 (0.92)** ^e^		0.53 (0.48)	
	Syntactic	0.84 (0.23)	**1.90 (1.16)^e^**		0.76 (0.44)	
	Acoustic	0.93 (0.15)	**2.19 (0.83)**		0.90 (0.47)	

Mean (standard deviation) values are shown; 12 trials were presented in each condition. Significant differences (*P* < 0.05) from healthy control values are indicated in bold; details of pair-wise comparisons between participant groups and deviant conditions are presented in Supplementary Tables 2 (*d*-prime data), 3 and 4 (pupil data), and Supplementary Tables 5 and 6 (IC correlation data) in Supplementary material.

AD, patient group with typical Alzheimer’s disease; bvFTD, patient group with behavioural variant frontotemporal dementia; Controls, healthy control group; IC, deviant information-content (see text); NA, not applicable; nfvPPA, patient group with non-fluent-agrammatic variant primary progressive aphasia; svPPA, patient group with semantic variant primary progressive aphasia.

a For no-deviant condition, values refer to correctly rejected trials (1—false-alarm rate), thus a reduced deviant detection accuracy (hit rate) in this condition signifies a raised false-alarm rate.

b In arbitrary units, for reference only.

c Significantly different from null hypothesis (no correlation).

d Significantly lower than nvfPPA group.

e Significantly lower than AD group.

There were significant main effects on performance (*d*-prime) of participant group [*χ*^2^(4) = 20.4, *P* < 0.001] and deviant condition [*χ*^2^(2) = 127.36, *P* < 0.001] and a significant interaction between group and condition [*χ*^2^(8) = 33.3, *P* < 0.001].

Comparing each patient group with the healthy control group, the bvFTD, svPPA and nfvPPA groups were significantly less sensitive in detecting semantic deviants (all *P* < 0.001) and syntactic deviants (all *P* < 0.005) and the nfvPPA group was additionally less sensitive in detecting acoustic deviants (*P* = 0.031), reflecting excessive false-alarms (Table 2). Comparing between patient groups, the bvFTD and svPPA groups were significantly less sensitive than the Alzheimer’s disease group in detecting syntactic deviants (all *P* < 0.042); while the nfvPPA group was significantly less sensitive than the Alzheimer’s disease group in detecting both syntactic deviants (*P* = 0.013) and semantic deviants (*P* = 0.045).

Comparing experimental conditions within participant groups, all groups were significantly more sensitive in detecting syntactic and acoustic deviants than semantic deviants (all *P* < 0.05). The bvFTD and svPPA groups were additionally significantly more sensitive in detecting acoustic than syntactic deviants (all *P* < 0.001).

Overall auditory deviant discriminability (*d*-prime) across the patient cohort correlated with musical background (*r* = 0.31, *P* = 0.01), pitch direction discrimination score (*r* = 0.39 = 0, *P* = 0.003), musical expertise score (rho = 0.31, *P* = 0.012) and musical familiarity *d*-prime (rho = 0.53, *P* < 0.001). There was no correlation of *d*-primes with age, gender, symptom duration, MMSE or peripheral hearing score.

Results of the ROC analysis with corresponding AUC coefficients are presented in Supplementary Fig. 3. Detection of both syntactic and semantic deviants discriminated well between each FTD syndromic group and healthy controls (AUC 0.81–0.87). No significant AUC differences were found between deviant conditions.

### Pupillometric data: autonomic responses to deviants in melodies

Participant groups did not differ significantly in resting baseline pupil size [*χ*^2^(4) = 6.6, *P* = 0.16] or overall pupil dynamics (mean pupil response across all trials) [*χ*^2^(4) = 8.2, *P* = 0.09].

**Figure 2:**
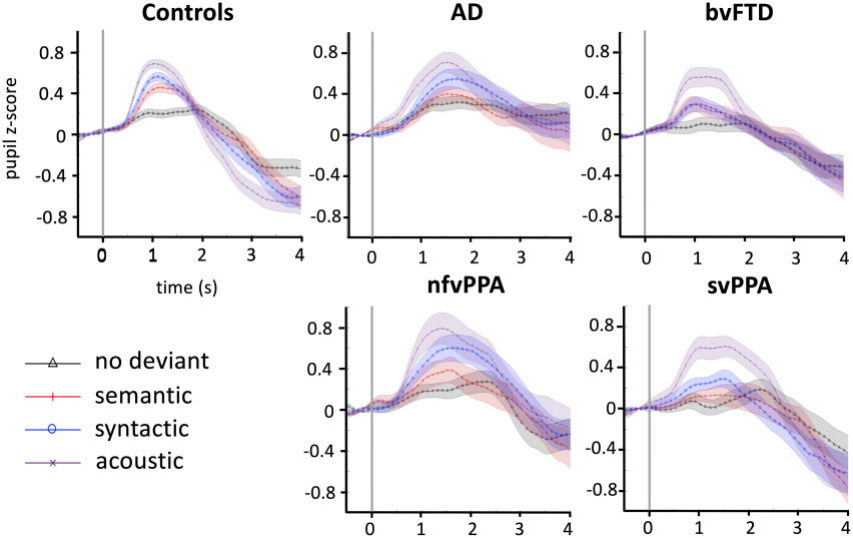
**Time course of pupil dilatation responses to melodic deviants in each of the experimental conditions, for all participant groups.** Onset of the deviant note is at time 0 (indicated by vertical grey line on each panel). To generate these pupil time series, trial-by-trial pupil time series from individual participants were first filtered, smoothed, converted to *z*-scores based on the signal mean and standard deviation for that participant’s dataset and baseline-corrected by subtracting the pre-deviant baseline (details in Supplementary material); the plots show the mean normalized pupil time series flanked by error envelopes representing the standard deviation of the group pupillary response, for each experimental condition (coded at lower left). AD, patient group with Alzheimer’s disease; bvFTD, patient group with behavioural variant frontotemporal dementia; Controls, healthy control group; nfvPPA, patient group with non-fluent-agrammatic variant primary progressive aphasia; svPPA, patient group with semantic variant primary progressive aphasia.

Group data for pupillary responses to melody deviants are summarized in Table 2 and Supplementary Tables 3 and 4; mean time courses of pupillary dilation responses are shown in Fig. 2 and Supplementary Fig. 4. There were significant main effects on mean pupillary response magnitude of participant group [*χ*^2^(4) = 10.3; *P* = 0.03] and deviant condition [*χ*^2^(3) = 123.18; *P* < 0.001] but no interaction [*χ*^2^(12) = 13.8; *P* = 0.31].

*Post-hoc* tests revealed that the nfvPPA and Alzheimer’s disease groups had significantly larger pupillary responses than the svPPA and bvFTD groups (all *P* < 0.05). There were no significant differences between any patient group and healthy controls (see Supplementary Table 3). When comparing conditions for all groups combined, there were significant differences between all types of deviants with pairwise comparisons preserving the following order: acoustic deviant > syntactic deviant > semantic deviant > no-deviant (all *P* < 0.001; see Supplementary Table 4).

### Information-theoretic modelling: processing statistical structure of melodies and deviants

Across the combined stimulus set, IC was positively correlated with deviant detection accuracy in the healthy control group (rho = 0.41, *P* = 0.01) and nfvPPA group (rho = 0.40, *P* = 0.02), whereas IC was negatively correlated with deviant detection accuracy in the svPPA group (rho = −0.43, *P* = 0.008). Correlations were significantly stronger in healthy controls and nfvPPA groups than both the bvFTD and svPPA groups (all *P* < 0.009; see Table 2, Supplementary Fig. 5 and Table 5). We found that for low ENT (more predictable) melodies, IC was significantly positively correlated with deviant detection accuracy in the healthy control group (rho = 0.52, *P* = 0.03), whereas IC was significantly negatively correlated with deviant detection accuracy in the svPPA group (rho = −0.57, *P* = 0.01), driven by a very low false-alarm rate in the no-deviant condition (see also Table 2). For high ENT (less predictable) melodies, there were no significant correlations between IC and deviant detection accuracy in any participant group.

Across the combined stimulus set, IC was positively correlated with pupillary dilatation magnitude in the healthy control group (rho = 0.56, *P* < 0.001), Alzheimer’s disease group (rho = 0.53, *P* < 0.001) and nfvPPA group (rho = 0.54, *P* < 0.001). The correlation was significantly stronger in healthy controls than the svPPA group (*P* = 0.04) and the bvFTD group (*P* = 0.04; see Table 2, Supplementary Fig. 5 and Table 6). For low ENT melodies, IC was significantly positively correlated with pupillary dilatation magnitude in the healthy control group (rho = 0.58, 0.01) and Alzheimer’s disease group (rho = 0.71, *P* < 0.001). For high ENT melodies, IC was significantly positively correlated with pupillary dilatation magnitude in the healthy control group (rho = 0.58, *P* = 0.01) and nfvPPA group (rho = 0.63, *P* = 0.01).

### Neuroanatomical associations

**Figure 3:**
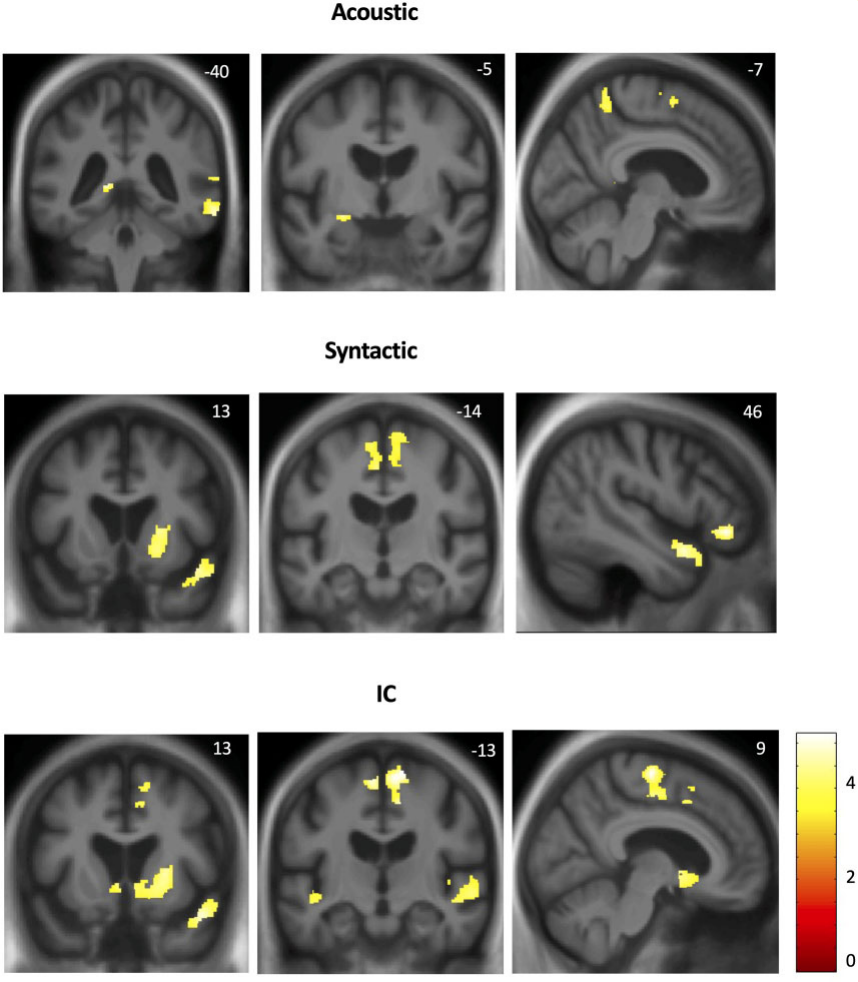
**Neuroanatomical correlates of accurate detection of melodic deviants and coupling between detection and musical surprise value in the combined patient cohort.** Statistical parametric maps (SPMs) show regional grey matter volume positively associated with acoustic (top panels) and syntactic (middle panels) deviant detection accuracy and coupling between deviant detection accuracy and deviant note IC (bottom panels) in familiar melodies, based on voxel-based morphometry of patients’ brain MR images. SPMs are thresholded for display purposes at *P* < 0.001 uncorrected over the whole brain; however, local maxima of areas shown were each significant at *P* < 0.05 after family-wise error correction for multiple voxel-wise comparisons within pre-specified anatomical regions of interest (see Table 3 and Supplementary Fig. 2); *T*-scores are coded on the colour bar. SPMs are overlaid on sections of the normalized study-specific T1-weighted mean brain MR image; the MNI coordinate (mm) of the plane of each section is indicated, and the right hemisphere is shown on the right in coronal sections.

Significant grey matter associations of deviant detection accuracy and coupling between deviant detection accuracy and deviant note IC for the combined patient cohort are summarized in Table 3; statistical parametric maps of these associations are presented in Fig. 3. All associations here are reported thresholded at *P* < 0.05 after FWE correction for multiple voxel-wise comparisons within pre-specified anatomical regions of interest.

**Table 3:** Neuroanatomical correlates of melodic deviant detection and surprise coding in the combined patient cohort

Condition	Region	Side	Cluster (voxels)	Peak (mm)			*T* score	P_FWE_
				*x*	*y*	*z*		
Acoustic	Precuneus	R	471	9	−46	67	4.94	0.022
		L	451	−10	−50	57	4.48	0.028
	Hippocampus	L	94	−14	−40	4	4.66	0.032
	Middle temporal gyrus	R	1489	64	−40	−14	4.59	0.022
	Supplementary motor area	L	75	−5	2	62	4.46	0.031
	Amygdala	L	124	−27	−6	−12	4.19	0.039
Syntactic	Supplementary motor area	R	982	4	−2	50	5.13	0.004
		L	1352	−4	−16	51	4.92	0.005
	Inferior frontal gyrus: pars orbitalis	R	488	50	38	−8	4.79	0.014
	Putamen	R	121	22	20	−6	4.61	0.006
	Temporal pole	R	1807	46	10	−20	4.51	0.015
IC correlation	Supplementary motor area	R	1076	14	−14	69	4.85	0.005
		L	945	−8	−16	62	4.84	0.007
	Superior temporal gyrus	L	1949	−56	−30	0	4.85	0.018
		R	2489	56	−16	−2	4.25	0.045
	Temporal pole	R	2489	46	9	−22	4.64	0.007
	Putamen	R	2883	22	20	−4	4.57	0.002
	Nucleus accumbens	L	1657	−4	10	−3	4.11	0.038
		R	2883	9	4	−8	4.08	0.018

The table presents the results of the voxel-based morphometry analysis. Shown are the locations of regional grey matter positively associated with accuracy of detection for different types of deviants and for the coupling between deviant detection accuracy and deviant note IC (individual Spearman rho) in familiar melodies, over the combined patient cohort (see also text and Fig. 3). Coordinates of local maxima are in standard MNI space. *P* values were all significant (*P* < 0.05) after family-wise error (FWE) correction for multiple voxel-wise comparisons within pre-specified anatomical regions of interest (see text and Supplementary Fig. 2).

Syntactic deviant detection accuracy correlated with regional grey matter in bilateral supplementary motor area and right pars orbitalis of inferior frontal gyrus, temporal pole and putamen. Acoustic deviant detection accuracy correlated with regional grey matter in a more postero-ventral, bi-hemispheric network including right precuneus and middle temporal gyrus, hippocampus and amygdala as well as left supplementary motor area. No associations of semantic deviant detection accuracy were identified at the prescribed significance threshold.

Coupling between deviant detection accuracy and deviant note IC (individual Spearman rho) correlated with regional grey matter in an extended bi-hemispheric network comprising bilateral supplementary motor area, superior temporal gyrus and nucleus accumbens and right temporal pole and putamen.

## Discussion

Here, we have shown that canonical syndromes of FTD and Alzheimer’s disease have differentiated behavioural and autonomic responses to musically surprising events. Consistent with previous evidence,^60^^,^^93–95^ healthy older controls here showed a graded response profile with more accurate detection of ‘surprising’ deviations in fundamental acoustic structure and generic syntactic musical rules than deviations in the semantic structure of specific musical objects (melodies). Further, in healthy controls, both detection accuracy and autonomic reactivity correlated with the information-theoretic quantity of surprise (IC) in deviant musical events but were differentially modulated by the predictability (ENT) of the musical environment. In line with previous work,^96^^,^^97^ detection accuracy was enhanced in more predictable musical environments, whereas pupillary response was not affected by environmental predictability. Whereas the Alzheimer’s disease group detected musical deviants normally, the bvFTD and svPPA groups showed strikingly similar profiles of impaired syntactic and semantic deviant detection and impaired cognitive and autonomic sensitivity to deviant IC, relative both to healthy controls and other syndromic groups; while the nfvPPA group showed a generalized deficit of auditory deviant discriminability but retained sensitivity to IC. These syndromic signatures were evident after taking elementary pitch pattern perception and musical experience into account. Across the patient cohort, detection of musical and acoustic deviants and the linkage between deviant detection accuracy and surprise had separable neuroanatomical substrates in cortico-subcortical networks previously implicated in the perceptual, semantic and hedonic analysis of music and in tracking probabilistic information in musical sequences.

Behaviourally, impaired detection of musical ‘rule’ violations in bvFTD and svPPA accords with previous evidence that these syndromes impair psychological expectations about melodies,^45^^,^^46^^,^^49^ auditory scenes^6^ and other complex sensory signals.^1^^,^^5^^,^^7–11^ Such deficits are in turn likely to underpin the difficulties these patients experience in interpreting the often ambiguous or conflictual emotional and social signals of other people and in regulating their own socio-emotional behaviours.^2^^,^^98–102^ In predictive coding terms, such phenomena might reflect impaired matching of incoming signals to stored neural ‘templates’ (predictions established through past experience), inefficient updating of those predictions and/or degraded templates *per se*. While any of these may operate in bvFTD and svPPA, the correlation here between detection accuracy and melody recognition (familiarity *d*-prime) underlines how an ‘incorrect model’ of the current sensory environment precludes detection of incongruent events. In bvFTD and svPPA, environmental deviations are essentially rendered ‘unsurprising’, while false-alarm rates (most strikingly, in svPPA) are correspondingly low (Table 2): this pattern would follow if stored melody templates are degraded to the extent that most incoming facsimiles of the template (including aberrant ones) achieve a ‘match’. Loss of the normal dependence of deviant detection on surprise value (IC) and lack of any modulatory effect from environmental predictability (ENT) in bvFTD and svPPA are consistent with previous evidence that svPPA fundamentally impairs analysis of the IC of auditory sequences^50^^,^^51^ while bvFTD impairs stimulus salience coding,^45^^,^^101^ as determined by the interaction of event surprise and environmental predictability.^103^^,^^104^

Our finding of a generalized abnormality of musical and acoustic deviant discriminability in nfvPPA builds on emerging evidence for disordered auditory processing in this syndrome.^50^^,^^66^^,^^105–107^ In contrast to other dementia syndromes (and mirroring the profile in svPPA), nfvPPA was associated with an abnormally high false-alarm rate (Table 2), indicating that these patients tended to over-interpret variations from musical canonicity in melodies without deviant notes (for example, timbral or key changes associated with transcribing the melodies) as ‘errors’. This abnormally heightened ‘surprise’ sensitivity is consistent with a previous predictive-coding account of degraded speech processing in this syndrome,^52^ according to which patients with nfvPPA tend to make inflexible predictions about incoming auditory data. In patients’ daily lives, reduced predictive flexibility under dynamic listening conditions may contribute to a range of deficits, including impaired hearing even in quiet environments, impaired perception of less familiar accents and emotional prosody and reduced modulation of social signals, such as conversational laughter.^106–109^

The reduced coupling between pupillary response and deviant IC in the svPPA and bvFTD groups here extends the evidence for central autonomic dysregulation in these syndromes.^63,110–115^ The syndromic pupillary response profiles here, taken together with the behavioural data on detection accuracy, suggest that coding of musical salience was deficient in patients with bvFTD and svPPA. A mutual interplay between prediction formation and salience coding, and between cognitive and autonomic mechanisms, is essential to our normal experience of musical events.^44^^,^^116^^,^^117^ Peak pupillary responses in our nfvPPA group tracked deviant surprise value in relatively less (but not more) predictable musical environments, as would be anticipated if pupillary responses in this syndrome tend to signal relatively less salient events linearly but become saturated in more predictable environments.^79^

Neuroanatomically, detection of acoustic deviants correlated with grey matter in middle temporal gyrus, precuneus and hippocampus: key components of the so-called ‘default-mode’ network that governs the interface between self and environment, facilitates reallocation of attention and more particularly, has been shown to play a key role in auditory scene analysis, both in the healthy brain and in neurodegenerative disease.^118^^,^^119^ Noise bursts interrupting a melody potentially signal a fundamental change in the prevailing auditory environment, engaging neural mechanisms that decode the significance of environmental fluctuations in relation to the internal milieu and the neural record of continuous auditory experience. Connected brain regions mediate the preparation of responses to such salient, potentially behaviourally relevant events: semantic control processes that integrate incongruous sensory information with stored conceptual representations are mediated via middle temporal gurus^120–122^ while physiological arousal is mediated via limbic structures, including amygdala.^123^^,^^124^

Detection of syntactic deviants and coupling of detection to deviant surprise value had closely overlapping neuroanatomical correlates subsuming a generic fronto-striatal ‘prediction network’ previously proposed to test sensory hypotheses and minimize prediction error in diverse domains, including music.^125^ Our findings are consistent with a scheme in which the prediction network is hierarchically engaged with involvement of additional regions mediating different levels of musical surprise analysis. According to this scheme, superior temporal gyrus initially represents musical object structure (especially, pitch pattern) and IC.^43^^,^^126–130^ Temporal polar cortex and pars orbitalis of inferior frontal cortex together track and evaluate incoming musical patterns against stored templates and rules acquired implicitly through the individual’s cumulative past experience of music.^55^^,^^131^^,^^132^ The temporal pole hosts the canonical ‘hub’ of the semantic memory system, while pars orbitalis integrates semantic and affective signals across sensory modalities and mediates subjective experience of expectation violations in music.^70^^,^^133^^,^^134^ Both regions are implicated in the recognition of melodies^49^^,^^65^^,^^91^^,^^135–138^ and in processing violations of musical semantic representations.^139^ The conjoint involvement of dorsal and ventral striatum here underlines the critical role of striatal dopaminergic circuitry in coding musical expectation and surprise probabilistically,^43^^,^^44^ an operation integral to hedonic valuation of music.^37^^,^^88^^,^^117^ Nucleus accumbens tracks reward prediction errors in music, a prime mover of musical learning and behaviour.^87^ While we did not assess reward prediction explicitly in this study, ventral striatum is engaged in resolving musical uncertainty through the integration of cognitive and affective information.^44^^,^^87^ By employing highly familiar melodies, this study may have primed the implicit coding of musical reward potential by striatal circuitry.

A common correlate of musical and acoustic surprise processing was identified in supplementary motor cortex. This is a core effector region for predicting actions and preparing behavioural responses to salient and arousing events, in music, vocalizations and other cognitive domains.^65^^,^^92^^,^^137^^,^^140–142^ This region is intimately linked to the salience network^90^ and plays an essential role in processing auditory expectations, especially when these have been established through sensorimotor integration as is generally the case for music.^92^^,^^143^^,^^144^

This study suggests that major dementias have distinct profiles of sensory ‘surprise’ processing, as instantiated in music. This is a fundamental cognitive and pathophysiological signal that applies a unifying approach to understanding the pathophysiology of complex behavioural changes in these diseases, suggests musical ‘tools’ to measure such changes and might inform future experimental work. However, the study raises a number of issues. The findings should be corroborated in larger patient cohorts and other neurodegenerative pathologies, and the basis for individual variability—a substantial factor within syndromic profiles (see Fig. 1)—and the longitudinal evolution of deficits require elucidation, particularly if stratification of syndromic groups is to yield novel disease biomarkers. The musical paradigm could be elaborated in various ways, including manipulation of prior melody familiarity (the listener’s prior expectations about the musical environment). Accumulating evidence that music processing shares neurobiological circuitry with the processing of social and emotional signals, in health as well as neurodegenerative disease,^42^^,^^53^^,^^54^^,^^145^ lends credence to our proposal that music may be a pertinent model system for deconstructing the neural mechanisms that underpin the wider phenotypic repertoire of FTD and Alzheimer’s disease. This is an exciting prospect, given that we currently largely lack experimental paradigms to assess social cognition conveniently, flexibly and cross-linguistically in cognitively impaired people.

The predictive coding formulation we have adopted in this study goes beyond neuroanatomical convergence, in suggesting a generic pathophysiological framework that potentially extends across different scales of description (from local neural circuits to the whole brain) and modalities (cognitive, autonomic and neuroimaging). However, the validity and utility of the predictive coding framework will only be established through the application of physiological and computational techniques—such as magnetoencephalography and dynamic causal modelling—that can capture neural microcircuits and large-scale network dynamics directly. Our findings suggest that music is an attractive candidate target for the further application of such techniques and the extraction of novel pathophysiological metrics of neurodegenerative disease. The potential explanatory power of the predictive coding paradigm in deconstructing complex symptoms has gained considerable traction in clinical psychiatry.^146–148^ However, it will be crucial to demonstrate that deficits of musical predictive coding are relevant to the socio-emotional symptoms reported by patients and caregivers, if this paradigm is ultimately to yield novel psychophysiological tools to detect and measure disease activity and strategies to ameliorate the effects of neurodegenerative proteinopathies.

## Supplementary material

Supplementary material is available at *Brain Communications* online.

## Supplementary Material

fcab173_Supplementary_Data

## Abbreviations

AUC =: area under the curve
bvFTD =: behavioural variant frontotemporal dementia
ENT =: entropy
FTD =: frontotemporal dementia
FWE =: family-wise error
IC =: information-content
MMSE =: Mini-Mental State Examination
nfvPPA =: non-fluent-agrammatic variant primary progressive aphasia
ROC =: receiver-operating-characteristic
svPPA =: semantic variant primary progressive aphasia
VBM =: voxel-based morphometry
