# Supplementary material for: Decoding expectation and surprise in dementia: the paradigm of music
Source: Brain Commun. 2021 Aug 10;3(3):fcab173. doi: 10.1093/braincomms/fcab173 (PMC8376684; doi:10.1093/braincomms/fcab173)
Supplement: fcab173_Supplementary_Data [file fcab173_supplementary_data.docx]

**Supplementary Material. Decoding expectation and surprise in dementia: the paradigm of music,**

**by E Benhamou et al.**

# Assessment of peripheral hearing function

Using an Otovation Roto® audiometer (https:/www.auditdata.com/) with a single TDH-39P 10-ohm Telephonics® earphone (www.telephonics.com) in a quiet room, steady tones of 500, 1000, 2000, 4000 and 6000Hz were presented separately to each of the participant’s ears, over ascending intensity levels commencing at 20 dB HL (decibel hearing level). At each frequency, the participant indicated (verbally or by gesture) when they first heard a noise. If the participant was unable to hear the tone, the level was increased in 5 dB increments (maximum 70dB HL). This procedure was repeated three times to establish the mean threshold at that frequency. For each participant, a composite score was created by calculating the mean threshold across all frequencies in the best ear.

# Assessment of pitch change direction processing

To provide a measure of elementary pitch pattern processing required for the perception of melodies, we adopted a procedure similar to those originated by Seashore (Seashore, 1919). This task was designed as a control task to determine whether participants had any deficit of elementary pitch change perception. We assessed participants’ ability to discriminate pitch direction changes in sequentially presented note pairs; the notes comprising each pair differed in pitch by one to five semitones. Individual notes comprising each pair had piano timbre and duration 1 second with inter-note gap 1 second. Ten trials (pairs) were presented and the direction of the pitch shift between notes in each pair was varied randomly across trials (five ascending, five descending). The task on each trial was to decide if the second note of the pair was ‘higher’ or ‘lower’ than the first note.

# Stimulus presentation and responses

All participants were first familiarised with the experiment and given several practice trials (using melodies not presented in the main experiment) to ensure they understood and could comply with the procedure. The participant was seated approximately 50 cm from a desktop computer monitor in a dimly and uniformly illuminated, quiet room, fixating a small white circle in the centre of the monitor screen. Each experimental trial comprised an initial brief silent baseline interval (2 s), followed by the melody stimulus and a final silent equilibration interval (varied randomly between 2 and 4 s). Stimuli were presented in randomised order from a notebook computer running Experiment Builder® (www.sr-research.com/experiment-builder) binaurally via headphones (Audio-Technica®) with an HD Digital 7.1 USB Audio Box at a constant, comfortable listening level (at least 70 dB). On completion of the post-stimulus equilibration interval (to avoid any confounding effects of online decision-making or motor processes on pupil dilatation), the task on each trial was to decide whether the melody contained a wrong note; the participant responded verbally or by pointing to the word ‘Yes’ or ‘No’ presented on the monitor.

Following the pupillometry session, we conducted a test to assess recognition of the stimulus melodies. Each participant was asked to listen to the famous melodies presented during the pupillometry session in canonical form (from the beginning of the melody to the time of onset of any deviant in the main experiment) alongside 24 unfamiliar (novel) melodies. Unfamiliar melodies were either pseudo-reversed versions of the familiar melodies or tunes composed de novo by an experienced musician (EB); the set of unfamiliar melodies was closely matched to the familiar melody set for loudness, duration, key, pitch range and tempo. The task on each trial was to indicate whether or not the melody was a well-known tune.

All participant responses were stored for offline analysis. No feedback about the performance was given during the pupillometry session or the melody recognition test session and no time limits were imposed.

# Pupil measurement and analysis

Pupil area was measured (sampling rate 500 Hz) from the right pupil using an infra-red camera [Eyelink II; SR Research, Canada] mounted on a headset just below the line of sight. Prior to data acquisition, a five-dot calibration was conducted to ensure adequate gaze measurements. Participants were asked to keep head movements to a minimum throughout the recording session and to restrict motion artefacts, the participant’s head was stabilised using the SR Research Head Support chinrest. Resting baseline pupil size was determined as the mean over the two-second silence interval before the start of each stimulus trial. Each stimulus trial was triggered by the experimenter when fixation was stable at less than two arbitrary gaze units away from the central fixation point.

In analysis, we first excluded all samples beyond three standard deviations of the mean signal for each participant and those with fixations directed at the periphery of the display screen (within 1 cm of the monitor border). Blinks were identified and removed from the signal using a procedure in MATLAB R2016b. Blinks were characterised by a rapid decline towards zero from blink onset, and a rapid rise back to the regular value at blink offset; 100ms of signal was removed before and after the missing data points and missing data were interpolated (four equally spaced time points were used to generate a cubic spline fit to the missing time points between blink onset (t2) and blink offset (t3) of the unsmoothed signal, with t1= t2-t3+t2 and t4= t3-t2+t3 (Mathôt *et al.*, 2018). Data were then band-pass filtered (low cut-off = 1Hz, high cut-off = 1/128 Hz) to remove slow fluctuations and smooth the signal (Kret and Sjak-Shie, 2019). To allow for comparisons across trials and participants, data for each participant were z-scored based on the signal mean and standard deviation computed across that participant’s dataset. Epochs (spanning 0.5 sec before the onset of deviant to four seconds after deviant onset) with more than 50% missing data were excluded from further analysis. Finally, z-scored data were baseline-corrected on a trial-by-trial basis by subtracting the z-scored mean pupil size over the 500 ms time window before deviant onset. This normalised pupil diameter was time-domain-averaged across trials for each condition.

# The Information Dynamics Of Music Model (IDyOM) (Pearce, 2005)

Pearce et al. (Pearce, 2005) built IDyOM based on music informatics, statistical language modelling and data compression techniques. IDyOM provides reliable computational measures of musical pitch unexpectedness or ‘surprise’ (as represented by information-content, IC) and uncertainty (as represented by entropy, ENT) for Western listeners (Pearce *et al.*, 2010; Omigie *et al.*, 2012; Pearce and Wiggins, 2012; Egermann *et al.*, 2013; Hansen and Pearce, 2014; Sauvé *et al.*, 2018). IDyOM has also successfully predicted several electrophysiological measures of expectation violation (Carrus *et al.*, 2013; Omigie *et al.*, 2013; Quiroga-Martinez *et al.*, 2019) and even psychophysiological and subjective emotional responses (Egermann *et al.*, 2013; Sauvé *et al.*, 2018).

To index musical expectations, IDyOM models both dynamic local learning of repeated patterns within stimuli (short-term or ‘online’ listening) and stylistic or rule-based learning through lifelong exposure to a large corpus of musical sequences (long-term listening). IC and ENT can be computed using different musical features (or viewpoints) as inputs to IDyOM: one could model the probability of the next pitch, registral direction, time, inter-onset interval ratio, etc., and one could model these different ‘viewpoints’ independently or simultaneously. Based on empirical findings concerning the integration of pitch information in melodies from pitch intervals and scale degrees (Pearce and Müllensiefen, 2017), in this study we chose two viewpoints as inputs to IDyOM: ‘cpitch’ (representing chromatic notes counted up and down from the middle pitch number, C=60) and ‘cpintfref cpint’ (representing chromatic pitch interval combined with scale degree – the chromatic interval from tonic, where 0 = tonic, 4 median, 7 dominant, etc.).

As a proxy for long-term listening (participants’ life-long exposure to Western tonal music), the model was trained on a large, representative sample of Western tonal music. We trained IDyOM on a corpus of 449 Western tonal melodies, comprising 43 American tunes, 71 English tunes, 150 German folk songs from the Essen folk collection (Schaffrath, 1992), and 185 chorale melodies harmonized by Bach as in other applications of IDyOM (Omigie *et al.*, 2012; Egermann *et al.*, 2013; Hansen and Pearce, 2014; Quiroga-Martinez *et al.*, 2019). This corpus constituted the ‘training set’ which allowed IDyOM to learn the statistical structure of Western tonal melodies, via variable-order Markov modelling (Pearce, 2005), This training on general musical structures mimics the listener’s life-long exposure to music; the trained ‘long-term’ model, therefore, represents ‘musical syntax’, the ‘rules’ about tonal music that listeners automatically internalise through their accumulated past experience of music. When listening to individual pieces of music, listeners continuously update their expectations about how the piece will subsequently unfold; these expectations depend not only on the long-term context established by past musical experience but the short-term context established by that particular piece. As a proxy for such short-term (online) listening, we used a second, ‘short-term’ model which dynamically learned the statistical structure of each stimulus used in our experiment. The short-term and long-term models both produce distributions characterising each note as the melody unfolds, in information-theoretic terms.

In combining the short-term and long-term models, the distributions of note characteristics generated by each model are weighted according to probability distribution, such that the less probable model is discounted relative to the more probable model. The weighted combined model outputs two key information theoretic parameters: IC, corresponding to the actual degree of unexpectedness of the pitch of each note; and ENT, corresponding to the prior uncertainty about a pitch note based on the variability of previously experienced notes. In mathematical terms, IC corresponds to the base-2 negative log probability of that note ‘event’, which is inversely proportional to the probability of an event x_i_ $h\left( x_{i} | c \right)= -{log}_{2}\left( p\left( x_{i} | c \right) \right)$ with c referring to the preceding context (MacKay, 2003) and ENT corresponds to $H\left( c \right)=\sum_{i\in A} p\left( x_{i} | c \right) \times h\left( x_{i} | c \right)$ (maximal when all potential events xi are equally probable, with $p\left( x_{i} | c \right)=\frac{1}{n}$ where n equals the number of notes). In psychological terms, IC is a “backward-looking” metric reflecting the amount of information processing required given the past melody context (the degree of unexpectedness or surprise associated with an event, such as a musical note, for the listener); while ENT is a “forward-looking” metric reflecting the predictability of an upcoming note (the listener’s degree of uncertainty about the event).

The constituent melodies of our deviant conditions (‘standard’ (or no-deviant), syntactic deviants and semantic deviants) did not reveal any significant differences in mean ENT (one-way ANOVA with Bonferroni correction, F(3,44) = 1.51, p=0.22). As our melodies were rather similar in musical character (see Supplementary Table 1) and were chosen so that they were highly familiar to listeners, ENT range here was quite narrow. IC values in the low ENT category ranged from 0.38 to 14.1 with a median value of 4.05 while IC values in the high ENT category ranged from 0.75 to 13.2 with a median value of 5.10). There was no significant difference between these two sets (t=-0.67, p = 0.51). However, IC scores for syntactic deviants (mean=10.52 SD=2.26) were significantly greater than IC scores for ‘standard’ notes (mean=1.15 SD=0.62) (t=13.35, p<0.001) and IC scores for semantic deviants (mean=4.78 SD=1.75) (t=6.72, p<0.001). Similarly, IC scores for semantic deviants were significantly greater than IC scores for ‘standard’ notes (t=6.55, p<0.001). IC distributions for each experimental melodic deviant condition are presented in Supplementary Figure 1 below.

# Pupillometry analysis

The difference in time series for each condition was computed for each participant, and these time series were subjected to bootstrap resampling (10,000 iterations; with replacement) with family-wise error (FWE)-corrected cluster-size threshold p<0.05 (Bonferroni-corrected with number of pairwise comparisons conducted). Any significant differences in the pre-onset interval (1 second before onset of deviant) would be attributable to noise, and the largest number of consecutive significant samples’ pre-onset was used as the threshold for the statistical analysis for the entire epoch (-1 to 4 seconds). Significant time intervals are presented as colored horizontal bars below the pupil time series plots (Figure 2).

# Sensitivity-specificity (ROC) analysis

In order to assess how well experimental parameters (type of melodic deviant and deviant IC) were able to discriminate behaviourally between each patient group and healthy controls, we constructed receiver operating characteristic (ROC) curves whereby the discriminatory ability of conditions was quantified using the area under the curve (AUC) for each comparison (Hanley and McNeil, 1982). The logistic regression model used to determine AUC included the pitch discrimination score as covariate of no-interest (including other covariates from the original linear mixed models would increase the chance of overfitting). The ROC analysis results were interpreted as follows: AUC <0.70, low diagnostic accuracy; AUC in the range of 0.70–0.90, moderate diagnostic accuracy; and AUC ≥0.90, high diagnostic accuracy.

# Brain image acquisition and pre-processing

For 58 patients (20 bvFTD, 12 svPPA, 11 nfvPPA, 15 AD), T1-weighted volumetric brain MR images were acquired a sagittal 3-D magnetization-prepared rapid-gradient-echo T1-weighted volumetric brain MR sequence (echo time/repetition time/ inversion time 2.9/2000/850 msec, dimensions 256-256-208, voxel size 1.1-1.1-1.1 mm) was acquired on a Prisma 3T MRI scanner using a 64-channel phased-array head-coil. MRI scans were missing for 4 AD patients, 1 bvFTD patient and 1 nfvPPA patient (of the ‘missing’ cases, 4 had a cardiac pacemaker, 1 could not have MRI due to claustrophobia and 1 was rejected on account of excessive movement). Pre-processing of brain images was performed using the New Segment (Weiskopf *et al.*, 2011) and DARTEL (Ashburner, 2007) toolboxes of SPM12 (www.fil.ion.ucl.ac.uk/spm) under Matlab and following an optimised protocol (Ridgway *et al.*, 2008). Normalisation, segmentation and modulation of grey and white matter images were performed using default parameter settings and grey matter images were smoothed using a 6 mm full width-at-half-maximum Gaussian kernel. A study-specific template mean brain image was created by warping all bias-corrected native space brain images to the final DARTEL template and calculating the average of the warped brain images. Total intracranial volume was calculated for each patient by summing grey matter, white matter and cerebrospinal fluid volumes after segmentation of tissue classes.

**Supplementary Table 1.** Characteristics of experimental melody stimuli

| **Melody** | **Duration**  (ms) | **Onset**  (ms) | **Key** | **Tempo**  (bpm) | **Pitch range** | **Fam** | **ENT** | **Deviant characteristics** | | |
| --- | --- | --- | --- | --- | --- | --- | --- | --- | --- | --- |
|  |  |  |  |  |  |  |  | **Interval** | **Position*** | **IC*** |
| ***No deviant*** | | | | | | | | | | |
| Colonel Bogey March | 8500 | 6000 | Cmaj | 120 | E4 - E5 | 5.00 | 2.72 | NA | -2 | 1.81 |
| Edelweiss (The Sound of Music) | 7478 | 3000 | Cmaj | 120 | E4 - D5 | 4.75 | 3.39 | NA | -4 | 1.57 |
| Moon River | 8407 | 4498 | Cmaj | 90 | F4 - D5 | 3.67 | 3.15 | NA | -3 | 1.10 |
| Morning (Peer Gynt) | 7790 | 4813 | Dmin | 120 | D4 - D5 | 4.88 | 2.65 | NA | -4 | 0.59 |
| My Way | 6911 | 5681 | Fmin | 140 | C4 - A4 | 4.70 | 2.43 | NA | -2 | 0.74 |
| Peter’s Theme (Peter and the Wolf ) | 8478 | 5990 | Cmaj | 120 | C4 - E5 | 5.00 | 2.63 | NA | -3 | 2.41 |
| Star Wars Theme | 9636 | 5352 | Amin | 90 | C4 - E5 | 4.85 | 2.16 | NA | -4 | 0.39 |
| Summertime | 8088 | 5990 | Emaj | 120 | E4 - E5 | 4.63 | 3.09 | NA | -3 | 0.75 |
| Swan Lake ‘Theme’ | 8088 | 5753 | Gmin | 120 | D4 - D5 | 4.79 | 2.78 | NA | -3 | 1.83 |
| Symphony No. 40 (Mozart) | 7500 | 5739 | Amin | 90 | D4 - C5 | 5.00 | 2.30 | NA | -2 | 0.61 |
| William Tell Overture | 7253 | 5805 | Gmaj | 120 | D4 - D5 | 5.00 | 2.04 | NA | -2 | 1.38 |
| You Are My Sunshine | 10421 | 7000 | Gmaj | 120 | D4 - E5 | 4.92 | 2.57 | NA | -4 | 0.65 |
| ***Semantic deviant*** | | | | | | | | | | |
| Auld Lang Syne | 8443 | 6000 | Gmaj | 120 | D4 - E5 | 5.00 | 2.66 | 8 | -2 | 5.55 |
| Away In A Manger | 7372 | 4800 | Gmaj | 100 | D4 - E5 | 4.79 | 2.79 | 4 | -3 | 3.24 |
| All Things Bright And Beautiful | 7444 | 5000 | Cmaj | 120 | C4 - C5 | 5.00 | 2.76 | 6 | -3 | 6.18 |
| Frere Jacques | 8392 | 5000 | Gmaj | 120 | G4 - D5 | 5.00 | 2.98 | 3 | -4 | 2.40 |
| God Save The Queen | 9403 | 6500 | Gmaj | 120 | F4 - C5 | 5.00 | 2.59 | 5 | -3 | 8.06 |
| Hark! The Herald Angels Sing | 8152 | 6500 | Gmaj | 120 | D4 - D5 | 5.00 | 2.54 | 5 | -2 | 4.60 |
| Jerusalem | 6980 | 4888 | Gmaj | 90 | C4 - C5 | 5.00 | 2.98 | 7 | -2 | 5.09 |
| Jingle Bells | 8465 | 6000 | Gmaj | 120 | G4 - D5 | 5.00 | 2.39 | 3 | -2 | 3.51 |
| Lullaby (Brahms) | 6915 | 4000 | Cmaj | 120 | E4 - D5 | 4.42 | 2.92 | 3 | -4 | 4.25 |
| O Come All Ye Faithful | 8613 | 6000 | Gmaj | 120 | D4 - D5 | 4.83 | 2.89 | 3 | -3 | 2.19 |
| Que Sera, Sera | 8500 | 6140 | Gmaj | 90 | G4 - E4 | 4.42 | 2.66 | 9 | -4 | 7.19 |
| We Wish You A Merry Christmas | 6987 | 4012 | Gmaj | 120 | C4 - C5 | 5.00 | 2.81 | 3 | -4 | 5.12 |
| ***Syntactic deviant*** | | | | | | | | | | |
| Deck The Halls | 7000 | 5636 | Gmaj | 140 | G4 - D5 | 5.00 | 2.76 | 4 | -2 | 9.36 |
| Do-Re-Mi (The Sound of Music) | 6142 | 3440 | Gmaj | 140 | G4 - C5 | 5.00 | 3.19 | 4 | -3 | 6.09 |
| Fly Me To The Moon | 7652 | 5000 | Cmaj | 120 | E4 - C5 | 3.25 | 2.82 | 6 | -3 | 10.74 |
| For He’s A Jolly Good Fellow | 5550 | 3800 | Gmaj | 100 | D4 - C5 | 4.79 | 2.65 | 6 | -2 | 7.82 |
| Für Elise (Beethoven) | 7173 | 3524 | Amin | 120 | E4 - E5 | 4.96 | 2.80 | 4 | -4 | 8.12 |
| Hey Jude | 9500 | 5053 | Cmaj | 90 | D4 - C5 | 4.54 | 3.38 | 6 | -4 | 13.21 |
| Joy To The World | 7790 | 5231 | Cmaj | 120 | C4 - C5 | 4.70 | 2.83 | 5 | -3 | 12.61 |
| La Donna E Mobile (Rigoletto) | 8245 | 5582 | Gmaj | 140 | G4 - D5 | 4.88 | 2.72 | 4 | -4 | 11.13 |
| O Little Town Of Bethlehem | 10000 | 6873 | Gmaj | 90 | D4 - D5 | 4.92 | 2.69 | 4 | -3 | 14.04 |
| Rudolph The Red-Nosed Reindeer | 6655 | 4300 | Cmaj | 140 | E4 - E5 | 4.96 | 2.94 | 8 | -2 | 11.82 |
| Silent Night | 6082 | 4000 | Cmaj | 120 | E4 - B4 | 4.92 | 3.20 | 3 | -2 | 11.03 |
| When The Saints Go Marching In | 8198 | 5247 | Gmaj | 120 | G4 - E5 | 4.83 | 2.56 | 4 | -4 | 10.25 |
| ***Acoustic deviant*** | | | | | | | | | | |
| Autumn Leaves | 9718 | 4750 | Dmin | 120 | C4 - B4 | 3.70 | 2.86 | NA | -4 | NA |
| Greensleeves | 5749 | 3500 | Gmaj | 120 | D4 - C5 | 4.85 | 2.80 | NA | -2 | NA |
| London Bridge Is Falling Down | 10000 | 6500 | Gmaj | 120 | G4 - D5 | 4.96 | 2.51 | NA | -4 | NA |
| Mary Had A Little Lamb | 10000 | 5000 | Gmaj | 120 | G4 - D5 | 4.63 | 2.71 | NA | -3 | NA |
| Once In Royal David’s City | 6620 | 5000 | Gmaj | 120 | C4 - G4 | 4.96 | 2.61 | NA | -4 | NA |
| Singin’ In The Rain | 7449 | 6000 | Gmaj | 120 | D4 - D5 | 4.64 | 2.75 | NA | -3 | NA |
| Somewhere Over The Rainbow | 7500 | 4500 | Cmaj | 90 | C4 - C5 | 4.93 | 2.73 | NA | -3 | NA |
| Three Blind Mice | 10000 | 6000 | Gmaj | 120 | G4 - D5 | 4.85 | 2.76 | NA | -4 | NA |
| Tonight (West Side Story) | 8000 | 4125 | Cmaj | 90 | C4 - C5 | 4.67 | 2.73 | NA | -3 | NA |
| Twelve Days Of Christmas | 8640 | 6550 | Gmaj | 90 | D4 - E5 | 4.89 | 2.54 | NA | -2 | NA |
| We Three Kings | 5924 | 4000 | Gmaj | 120 | E4 - B4 | 4.88 | 2.88 | NA | -3 | NA |
| Yesterday | 9776 | 6000 | Cmaj | 90 | C4 - C5 | 4.54 | 2.70 | NA | -2 | NA |

The table lists each melody from the stimuli set, its key parameters and the condition in which it was used. The melodies were chosen based on the results of a survey involving 15 healthy older British individuals who did not form part of the experimental cohort. From an initial set of 150 melodies, 48 highly familiar melodies were selected based on mean familiarity rating >3.5 on a 5-point scale ranging from 1 (completely unfamiliar) to 5 (very familiar). The note sequences corresponding to each melody were synthesised with piano timbre as digital wavefiles using MuseScore®; acoustic deviants were created using the Gaussian white-noise function in Matlab and were fixed for loudness (rms intensity) to the rest of the melody. Stimuli were fixed for overall loudness (rms intensity) and varied in length between three and five bars. A deviant note was always positioned in the second half of the melody, in order to allow initial establishment of the melodic context, and occurred on an on-beat; a pitch deviant always violated the contour of the canonical melody (between three and nine semitones). The melody conditions did not differ significantly in mean length (one-way ANOVA with Bonferroni correction, F(3,44) = 0.98, p=0.41), pitch range (Kruskal-Wallis non parametric test and post-hoc two-sample Wilcoxon tests with Bonferroni correction, p>0.05), tempo (Kruskal-Wallis non parametric test and post-hoc two-sample Wilcoxon tests with Bonferroni correction, p>0.05), familiarity rating (Kruskal-Wallis non parametric test and post-hoc two-sample Wilcoxon tests with Bonferroni correction, p>0.05) or entropy (one-way ANOVA with Bonferroni correction, F(3,44) = 1.51, p=0.22) of constituent melodies. IC scores for syntactic deviants (mean=10.5 SD=2.3) were significantly greater than IC scores for semantic deviants (mean=4.8 SD=1.8) (t=-3.9, p<0.001). *values for equivalently positioned standards in the case of melodies with no deviant; ENT, mean melody entropy (see text for details); Fam, familiarity rating; IC, information-content of deviants; Interval, no. of semitones difference between original (standard) and deviant note; NA, not applicable; Onset, time of deviant onset from start of stimulus; Position, position of deviant in melody note sequence (coded as: -2, penultimate on-beat; -3, third from last on-beat; -4, fourth from last on-beat (equally distributed across conditions with four trials at each position); Tempo, beats per minute (bpm; values were set to 90, 120 or 140 bm to reduce overall tempo variability across the stimulus set while also allowing natural tempo variations inherent to the melodies).

**Supplementary Table 2.** Comparisons between participant groups and conditions for accuracy (dprimes) of melodic deviant detection

| **Group** | **Condition** | **Comparisons** | | | | | |
| --- | --- | --- | --- | --- | --- | --- | --- |
|  |  | **Between participant groups** | | | | **Between deviant conditions** | |
|  |  | AD | bvFTD | svPPA | nfvPPA | Semantic | Syntactic |
| **Controls** | Semantic | -0.55  [-0.9 0.03]  p = 0.07 | **-1.23**  **[-1.41 -0.49]**  **p < 0.001** | **-1.38**  **[-1.69 -0.47]**  **p = 0.001** | **-1.36**  **[-1.64 -0.48]**  **p < 0.001** |  |  |
|  | Syntactic | -0.22  [-0.63 0.30]  p = 0.48 | **-1.22**  **[-1.41 -0.49]**  **p < 0.001** | **-1.12**  **[-1.48 -0.26]**  **p = 0.005** | **-1.22**  **[-1.53 -0.37]**  **p = 0.001** | **-0.47**  **[-0.62 -0.13]**  **p = 0.003** |  |
|  | Acoustic | -0.19  [-0.62 0.31]  p = 0.53 | -0.14  [-0.57 0.35]  p = 0.64 | -0.06  [-0.66 0.56]  p = 0.87 | **-0.82**  **[-1.22 -0.06]**  **p = 0.03** | **-0.59**  **[-0.71 -0.22]**  **p < 0.001** | -0.11  [-0.34 0.16]  p = 0.47 |
| **AD** | Semantic |  | -0.67  [-1.04 0.002]  p = 0.05 | -0.83  [-1.32 0.03]  p = 0.06 | **-0.81**  **[-1.24 -0.01]**  **p = 0.04** |  |  |
|  | Syntactic |  | **-1.00**  **[-1.3 -0.25]**  **p = 0.04** | **-0.90**  **[-1.37 -0.02]**  **p = 0.04** | **-1.00**  **[-1.40 -0.17]**  **p = 0.013** | **-0.81**  **[-0.97 -0.30]**  **p < 0.001** |  |
|  | Acoustic |  | 0.05  [-0.48 0.57]  p = 0.88 | 0.13  [-0.57 0.78]  p = 0.77 | -0.61  [-1.10 0.13]  p = 0.12 | **-0.95**  **[-1.08 -0.41]**  **p < 0.001** | -0.13  [-0.44 0.23]  p = 0.53 |
| **bvFTD** | Semantic |  |  | -0.15  [-0.79 0.54]  p = 0.71 | -0.13  [-0.72 0.51]  p = 0.74 |  |  |
|  | Syntactic |  |  | 0.10  [-0.58 0.74]  p = 0.81 | -0.001  [-0.62 0.62]  p = 0.99 | **-0.49**  **[-0.69 -0.07]**  **p = 0.02** |  |
|  | Acoustic |  |  | 1.23  [-0.60 0.73]  p =0.85 | -0.68  [-1.15 0.09]  p = 0.09 | **-1.68**  **[-1.62 -0.99]**  **p < 0.001** | **-1.19**  **[-1.24 -0.61]**  **p < 0.001** |
| **svPPA** | Semantic |  |  |  | 0.03  [-0.73 0.77]  p = 0.96 |  |  |
|  | Syntactic |  |  |  | -0.10  [-0.84 0.67]  p = 0.83 | **-0.76**  **[-1.09 -0.09]**  **p = 0.02** |  |
|  | Acoustic |  |  |  | -0.75  [-1.34 0.16]  p = 0.12 | **-1.92**  **[-2.00 -0.99]**  **p < 0.001** | **-1.16**  **[-1.41 -0.41]**  **p < 0.001** |
| **nfvPPA** | Syntactic |  |  |  |  | **-0.63**  **[-0.93 -0.04]**  **p = 0.03** |  |
|  | Acoustic |  |  |  |  | **-1.14**  **[-1.34 -0.44]**  **p < 0.001** | -0.51  [-0.85 0.05]  p = 0.08 |

Open cells present Cohen’s d effect sizes (with [95% confidence intervals] and p-values) between d-prime values for the intersecting participant groups (for the melodic deviant condition corresponding to that row) or for the intersecting melodic deviant conditions (within the participant group corresponding to that row); significant pairwise comparisons (p < 0.05) are indicated in bold. AD, patient group with typical Alzheimer’s disease; bvFTD, patient group with behavioural variant frontotemporal dementia; Controls, healthy control group; nfvPPA; patient group with nonfluent-agrammatic variant primary progressive aphasia; svPPA, patient group with semantic variant primary progressive aphasia.

**Supplementary Table 3.** Mean maximum pupillary responses to melodic deviants: comparisons between participant groups

| **Group** | **Comparisons between participant groups** | | | |
| --- | --- | --- | --- | --- |
|  | **AD** | **bvFTD** | **svPPA** | **nfvPPA** |
| **Controls** | 0.24  [-0.06 0.33]  p = 0.18 | -0.31  [-0.24 0.05]  p = 0.19 | -0.46  [-0.34 0.04]  p = 0.12 | 0.46  [-0.03 0.34]  p = 0.10 |
| **AD** |  | **-0.52**  **[-0.34 -0.01]**  **p = 0.041** | **-0.70**  **[-0.44 -0.01]**  **p = 0.037** | 0.23  [-0.12 0.27]  p = 0.44 |
| **bvFTD** |  |  | -0.16  [-0.26 0.16]  p = 0.63 | **0.76**  **[0.06 0.45]**  **p = 0.012** |
| **svPPA** |  |  |  | **0.92**  **[0.06 0.54]**  **p = 0.013** |

Open cells present Cohen’s effect sizes (with [95% confidence intervals] and p-values) between magnitudes of mean peak pupil dilatation response for the intersecting participant groups (across all melodic deviant conditions); significant pairwise comparisons (p < 0.05) are indicated in bold. AD, patient group with typical Alzheimer’s disease; bvFTD, patient group with behavioural variant frontotemporal dementia; Controls, healthy control group; nfvPPA; patient group with nonfluent-agrammatic variant primary progressive aphasia; svPPA, patient group with semantic variant primary progressive aphasia.

**Supplementary Table 4.** Mean maximum pupillary responses to melodic deviants: comparisons between experimental conditions

| **Condition** | **Comparisons between deviant conditions** | | |
| --- | --- | --- | --- |
|  | No deviant | Semantic | Syntactic |
| Semantic | **-0.43**  **[-0.21 -0.06]**  **p < 0.001** |  |  |
| Syntactic | **-0.73**  **[-0.31 -0.17]**  **p < 0.001** | **-0.31**  **[-0.17 -0.03]**  **p = 0.006** |  |
| Acoustic | **-1.32**  **[-0.50 -0.36]**  **p < 0.001** | **-0.89**  **[-0.37 -0.22]**  **p < 0.001** | **-0.58**  **[-0.26 -0.12]**  **p < 0.001** |

Open cells present Cohen’s effect sizes (with [95% confidence intervals] and p-values) between magnitudes of mean peak pupil dilatation response for the intersecting melodic deviant conditions (across all participant groups); significant pairwise comparisons (p < 0.05) are indicated in bold.

**Supplementary Table 5.** Correlations of detection accuracy with deviant information-content: participant group values and comparisons

| **Group** | **Condition** | **IC correlate** | **Comparisons** | | | |
| --- | --- | --- | --- | --- | --- | --- |
|  |  |  | AD | bvFTD | svPPA | nfvPPA |
| **Controls** | All | **rho = 0.41** | p = 0.09  [-3.01 0.31] | **p = 0.008**  **[-4.69 -0.77]** | **p < 0.001**  **[-5.91 -1.99]** | p = 0.48  [-1.70 1.60] |
|  | low ENT | **rho = 0.52** | p = 0.18  [-1.86 1.44] | **p = 0.023**  **[-4.33 -0.41]** | **p < 0.001**  **[-5.73 -1.81]** | p = 0.37  [-1.98 1.32] |
|  | high ENT | rho = 0.27 | p = 0.14  [-2.67 0.63] | p = 0.10  [-2.91 0.39] | p = 0.06  [-4.53 0.61] | p = 0.37  [-1.03 2.27] |
| **AD** | All | rho = 0.10 |  | p = 0.14  [-2.71 0.59] | **p = 0.01**  **[-4.55 -0.63]** | p = 0.09  [-0.34 2.96] |
|  | low ENT | rho = 0.24 |  | p = 0.14  [-2.77 0.53] | **p = 0.007**  **[-4.79 -0.87]** | p = 0.28  [-1.04 2.26] |
|  | high ENT | rho = -0.11 |  | p = 0.42  [-1.83 1.52] | p = 0.32  [-2.09 1.21] | p = 0.11  [-0.45 1.85] |
| **bvFTD** | All | rho = -0.16 |  |  | p = 0.11  [-2.86 0.44] | **p = 0.009**  **[0.72 4.64]** |
|  | low ENT | rho = -0.15 |  |  | p = 0.09  [-2.05 0.25] | **p = 0.04**  **[0.08 3.38]** |
|  | high ENT | rho = -0.18 |  |  | p = 0.40  [-1.83 1.27] | p = 0.075  [-0.26 3.04] |
| **svPPA** | All | **rho = -0.43** |  |  |  | **p < 0.001**  **[1.94 5.86]** |
|  | low ENT | **rho = -0.57** |  |  |  | **p = 0.001**  **[1.48 5.40]** |
|  | high ENT | rho = -0.27 |  |  |  | **p = 0.045**  **[0.01 3.29]** |
| **nfvPPA** | All | **rho = 0.40** |  |  |  |  |
|  | low ENT | rho = 0.43 |  |  |  |  |
|  | high ENT | rho = 0.33 |  |  |  |  |

The table summarises correlations (Spearman’s rho) of melodic deviant information-content (IC) with detection accuracy (hit rate) for each participant group and p-values and 95% confidence intervals (after Fisher’s z transformations) on pair-wise comparisons of correlation values between groups. Data are shown for the combined stimulus melody set (All), melodies with low entropy (low ENT) and high entropy (high ENT), respectively (see main text and Supplementary Material above for background). Significant correlations and comparisons (p<0.05) are indicated in bold. AD, patient group with typical Alzheimer’s disease; bvFTD, patient group with behavioural variant frontotemporal dementia; Controls, healthy control group; nfvPPA; patient group with nonfluent-agrammatic variant primary progressive aphasia; svPPA, patient group with semantic variant primary progressive aphasia

**Supplementary Table 6.** Correlations of pupillary response magnitude with deviant information-content: participant group values and comparisons

| **Group** | **Condition** | **IC correlate** | **Comparisons** | | | |
| --- | --- | --- | --- | --- | --- | --- |
|  |  |  | AD | bvFTD | svPPA | nfvPPA |
| **Controls** | All | **rho = 0.56** | p = 0.43  [-2.13 1.79] | **p = 0.04**  **[-3.39 -0.09]** | **p = 0.044**  **[-3.35 -0.05]** | p = 0.45  [-1.53 1.77] |
|  | low ENT | **rho = 0.58** | p = 0.27  [-3.31 0.61] | p = 0.13  [-2.71 0.6] | p = 0.09  [-2.99 0.31] | p = 0.26  [-2.61 1.00] |
|  | high ENT | **rho = 0.58** | p = 0.21  [-3.53 0.39] | p = 0.13  [-2.79 0.51] | p = 0.11  [-2.85 0.45] | p = 0.41  [-1.43 1.86] |
| **AD** | All | **rho = 0.53** |  | p = 0.06  [-3.22 0.08] | p = 0.06  [-3.18 0.12] | p = 0.48  [-1.59 1.71] |
|  | low ENT | **rho = 0.71** |  | **p = 0.047**  **[-3.32 -0.02]** | **p = 0.025**  **[-3.61 -0.31]** | p = 0.10  [-2.92 0.38] |
|  | high ENT | rho = 0.35 |  | p = 0.37  [-1.98 1.32] | p = 0.35  [-2.04 1.26] | p = 0.15  [-0.62 2.68] |
| **bvFTD** | All | rho = 0.20 |  |  | p = 0.48  [-1.61 1.69] | p = 0.05  [-0.02 3.28] |
|  | low ENT | rho = 0.27 |  |  | p = 0.39  [-1.85 1.36] | p = 0.34  [-1.25 2.05] |
|  | high ENT | rho = 0.24 |  |  | p = 0.48  [-1.71 1.59] | p = 0.09  [-0.29 3.01] |
| **svPPA** | All | rho = 0.21 |  |  |  | p = 0.06  [-0.06 3.24] |
|  | low ENT | rho = 0.17 |  |  |  | p = 0.24  [-0.96 2.34] |
|  | high ENT | rho = 0.22 |  |  |  | p = 0.08  [-0.24 3.07] |
| **nfvPPA** | All | **rho = 0.54** |  |  |  |  |
|  | low ENT | rho = 0.40 |  |  |  |  |
|  | high ENT | **rho = 0.63** |  |  |  |  |

The table summarises correlations (Spearman’s rho) of melodic deviant information-content (IC) with maximum pupillary response for each participant group and p-values and 95% confidence intervals (after Fisher’s z transformations) on pair-wise comparisons of correlation values between groups. Data are shown for the combined stimulus melody set (All), melodies with low entropy (low ENT) and high entropy (high ENT), respectively (see main text and Supplementary Material above for background). Significant correlations and comparisons (p<0.05) are indicated in bold. AD, patient group with typical Alzheimer’s disease; bvFTD, patient group with behavioural variant frontotemporal dementia; Controls, healthy control group; nfvPPA; patient group with nonfluent-agrammatic variant primary progressive aphasia; svPPA, patient group with semantic variant primary progressive aphasia

Supplementary Figure 1. Information-content distributions for each melodic deviant condition (no deviant or ‘standard’; syntactic deviants; semantic deviants)

**Supplementary Figure 2.**  Pre-specified anatomical regions of interest for voxel-based morphometric analysis. Representative sagittal (A, B) and coronal (C, D) sections are shown for the neuroanatomical volumes selected for multiple voxel-wise comparison correction in the region-of-interest analyses based on prior anatomical hypotheses. These regions were customised from the Oxford/Harvard brain maps to fit the group mean template brain image. Regions comprise a posterior temporo-parietal network including posterior temporal gyrus, angular and supramarginal gyri and precuneus (cyan); an anteroventral network including temporal pole, anterior superior and middle temporal gyri and inferior frontal gyrus (red); a cingulo-insular network including anterior cingulate, supplementary motor area and insula (green); and a striato-limbic network including putamen, caudate nucleus, hippocampus and amygdala (white). Small volumes are rendered on sections of the mean normalised brain template for the patient cohort.

**Supplementary Figure 3.** ROC analysis indicating prediction of disease by performance detecting different types of melodic deviant, for each syndromic group vs health controls. Performance area under the curve (AUC) is indicated below the graph for each ROC curve. The ROC curves use d-primes for each deviant detection task to discriminate between patient groups and healthy controls. AD, patient group with Alzheimer’s disease; bvFTD, patient group with behavioural variant frontotemporal dementia; nfvPPA, patient group with nonfluent-agrammatic variant primary progressive aphasia; svPPA, patient group with semantic variant primary progressive aphasia.

**Supplementary Figure 4.** **Time course of pupil dilatation responses to melodic deviants in each of the experimental conditions, for all participant groups and results of bootstrap timeseries analysis to inform our choice of interval for extracting peak pupillary response.** Onset of the deviant note is at time 0. To generate these pupil time series, trial-by-trial pupil time series from individual participants were first filtered, smoothed, converted to z-scores based on the signal mean and standard deviation for that participant’s dataset and baseline-corrected by subtracting the pre-deviant baseline; the plots show the average normalised pupil diameter pupil time series. The shaded area shows ±1 SEM. Color-coded horizontal lines at graph bottom indicate time intervals where bootstrap resampling (10,000 iterations after family-wise error corrected cluster-size threshold p<0.05 and Bonferroni-corrected with number of pairwise comparisons conducted) confirmed significant differences between groups. AD, patient group with Alzheimer’s disease; bvFTD, patient group with behavioural variant frontotemporal dementia; Controls, healthy control group; nfvPPA, patient group with nonfluent-agrammatic variant primary progressive aphasia; svPPA, patient group with semantic variant primary progressive aphasia.

**Supplementary Figure 5.** Correlations (Spearman’s rho) of melodic deviant information-content (IC) with accuracy (hit rate) of deviant detection (top panels) and peak pupillary response (pupil max) to deviants (bottom panels), for each participant group. IC was estimated across the stimulus melody set using the IDyOM model (see text and Supplementary Material for details). In each panel, each dot represents one of the 36 stimulus melodies (for the matched no-deviant [black], semantic deviant [blue] and syntactic deviant [red] conditions; see text and Supplementary Table 1) and fitted lines indicate linear correlations between IC and the participant parameter of interest (N.B.: fitted lines are for reference only and are fitted to Pearson linear correlations which led to similar results than Spearman analysis). Open dots and solid lines code melody stimuli with low entropy (ENT); filled dots and dashed lines code melody stimuli with high entropy. Numerical and statistical data on the correlations are in Table 2 and Supplementary Tables 5 and 6; correlation (rho) values for the subsets of low ENT and high ENT melody stimuli and whether these were significant (sig; p<0.05) or non-significant (non sig) are also indicated on the panels for convenience . AD, patient group with Alzheimer’s disease; bvFTD, patient group with behavioural variant frontotemporal dementia; nfvPPA, patient group with nonfluent-agrammatic variant primary progressive aphasia; svPPA, patient group with semantic variant primary progressive aphasia.

**Supplementary references**

Ashburner J. A fast diffeomorphic image registration algorithm. Neuroimage 2007; 38: 95–113.

Carrus E, Pearce MT, Bhattacharya J. Melodic pitch expectation interacts with neural responses to syntactic but not semantic violations. Cortex 2013; 49: 2186–200.

Egermann H, Pearce MT, Wiggins GA, McAdams S. Probabilistic models of expectation violation predict psychophysiological emotional responses to live concert music. Cogn Affect Behav Neurosci 2013; 13: 533–53.

Hansen NC, Pearce MT. Predictive uncertainty in auditory sequence processing [Internet]. Front Psychol 2014; 5[cited 2018 Mar 5] Available from: https://www.frontiersin.org/articles/10.3389/fpsyg.2014.01052/full

Kret ME, Sjak-Shie EE. Preprocessing pupil size data: Guidelines and code. Behav Res 2019; 51: 1336–42.

MacKay DJC. Information Theory, Inference and Learning Algorithms. Cambridge University Press; 2003

Mathôt S, Fabius J, Van Heusden E, Van der Stigchel S. Safe and sensible preprocessing and baseline correction of pupil-size data. Behav Res Methods 2018; 50: 94–106.

Omigie D, Pearce MT, Stewart L. Tracking of pitch probabilities in congenital amusia. Neuropsychologia 2012; 50: 1483–93.

Omigie D, Pearce MT, Williamson VJ, Stewart L. Electrophysiological correlates of melodic processing in congenital amusia. Neuropsychologia 2013; 51: 1749–62.

Pearce M, Müllensiefen D. Compression-based Modelling of Musical Similarity Perception. Journal of New Music Research 2017; 46: 135–55.

Pearce MT. The construction and evaluation of statistical models of melodic structure in music perception and composition [Internet]. 2005[cited 2020 Sep 3] Available from: https://openaccess.city.ac.uk/id/eprint/8459/

Pearce MT, Ruiz MH, Kapasi S, Wiggins GA, Bhattacharya J. Unsupervised statistical learning underpins computational, behavioural, and neural manifestations of musical expectation. NeuroImage 2010; 50: 302–13.

Pearce MT, Wiggins GA. Auditory expectation: the information dynamics of music perception and cognition. Top Cogn Sci 2012; 4: 625–52.

Quiroga-Martinez DR, Hansen NC, Højlund A, Pearce MT, Brattico E, Vuust P. Reduced prediction error responses in high-as compared to low-uncertainty musical contexts. Cortex 2019; 120: 181–200.

Ridgway GR, Henley SMD, Rohrer JD, Scahill RI, Warren JD, Fox NC. Ten simple rules for reporting voxel-based morphometry studies. Neuroimage 2008; 40: 1429–35.

Sauvé SA, Sayed A, Dean RT, Pearce MT. Effects of pitch and timing expectancy on musical emotion. Psychomusicology: Music, Mind, and Brain 2018; 28: 17–39.

Seashore CE. The psychology of musical talent. New York, NY, US: Silver, Burdett & Company; 1919

Weiskopf N, Lutti A, Helms G, Novak M, Ashburner J, Hutton C. Unified segmentation based correction of R1 brain maps for RF transmit field inhomogeneities (UNICORT). Neuroimage 2011; 54: 2116–24.
